# Saccharpiscinols A–C: Flavans with Potential Anti-Inflammatory Activities from One Actinobacteria *Saccharomonospora piscinae*

**DOI:** 10.3390/molecules26164909

**Published:** 2021-08-13

**Authors:** Yung-Shun Su, Jih-Jung Chen, Ming-Jen Cheng, Chee-Yin Chai, Aij-Lie Kwan, Jheng-Cian Huang, Yueh-Hsiung Kuo

**Affiliations:** 1 Graduate Institute of Medicine, College of Medicine, Kaohsiung Medical University (KMU), Kaohsiung 807, Taiwan; mariussu@gmail.com (Y.-S.S.); ccjtsai@yahoo.com (C.-Y.C.); 2 Department of Dermatology, Kaohsiung Medical University Chung-Ho Memorial Hospital, Kaohsiung 807, Taiwan; 3 Department of Pharmacy, School of Pharmaceutical Sciences, National Yang Ming Chiao Tung University (NYCU), Taipei 112, Taiwan; jjungchen@nycu.edu.tw; 4 Department of Medical Research, China Medical University Hospital, Taichung 404, Taiwan; 5 Bioresource Collection and Research Center (BCRC), Food Industry Research and Development Institute (FIRDI), Hsinchu 300, Taiwan; 6 Department of Pathology, Kaohsiung Medical University Chung-Ho Memorial Hospital, Kaohsiung 807, Taiwan; 7 Ph.D. Program in Environmental and Occupational Medicine, College of Medicine, Kaohsiung Medical University and National Health Research Institutes, Kaohsiung 807, Taiwan; 8 Department of Neurosurgery, Kaohsiung Medical University Chung-Ho Memorial Hospital, Kaohsiung 807, Taiwan; 9 Department of Chemistry, National Taiwan University, Taipei 106, Taiwan; huangjc2007@gmail.com; 10 Department of Biotechnology, Asia University, Taichung 413, Taiwan; 11 Department of Chinese Pharmaceutical Sciences and Chinese Medicine Resources, College of Pharmacy, China Medical University, Taichung 404, Taiwan

**Keywords:** *Saccharomonospora piscinae*, pseudonocardiaceae, actinobacteria, secondary metabolites, NO inhibition

## Abstract

Phytochemical investigation and chromatographic separation of extracts from the actinobacteria strain *Saccharomonospora* *piscinae* that was isolated from dried fishpond sediment of Kouhu township, in the south of Taiwan, led to the isolation of three new compounds, saccharpiscinols A–C (**1**–**3**, respectively), and three new natural products*,* namely (2*S*)-5,7,3′,4′-tetrahydroxy-6,8-dimethylflavanone (**4**), methyl-4-hydroxy-2-methoxy-6-methylbenzoate (**5**), and (±)-7-acetyl-4,8-dihydroxy-6-methyl-1-tetralone (**6**). Compounds **4**–**6** were reported before as synthesized products, herein, they are reported from nature for the first time. The structures of the new compounds were unambiguously elucidated on the basis of extensive spectroscopic data analysis (1D- and 2D-NMR, MS, and UV) and comparison with literature data. The effect of some isolates on the inhibition of NO production in lipopolysaccharide-activated RAW 264.7 murine macrophages was evaluated. Saccharpiscinol A showed inhibitory activities against LPS-induced NO production.

## 1. Introduction

Actinobacteria are widely distributed in nature, and they are very useful in the pharmaceutical industry due to their seemingly unlimited capacity to produce secondary metabolites with diverse chemical structures and biological activities [1,2,3]. They are gram positive, free-living saprophytic bacteria that are found widely distributed in soil, water, and colonizing plants. Actinobacteria inhabitants have been identified as one of the major groups of the soil population [2,3], which may vary with the soil type. In the process of investigation on the diversity of cultivable actinobacteria associated with soil from Taiwan, we isolated a strain named 06168H-1^T^, which was isolated from a fishpond sediment sample collected from the southern area of Taiwan and had a unique morphology [4]. This strain was determined to be *Saccharomonospora piscinae* (Family: Pseudonocardiaceae), based on their phenotypic and genotypic data [4]. In order to find new bioactive compounds, the rare genera in this group have been identified and studied, including the genus *Saccharomonospora*, which is a genus of bacteria that was only discovered by Nonomura and Ohara, and contained 12 species until now [4]. *Saccharomonospora* strains are widely distributed in nature, including in soil, compost, plant bodies, marine sediments, sponges, and other environments. Therefore, in addition to mesophilic bacteria, there are also thermophilic strains. The spores of some thermophilic strains (*Saccharomonospora viridis*) can cause allergic pneumonia (farmer’s lung disease). In addition, research reports indicate that strains of this genus can decompose many natural or synthetic compounds, such as polyester, rice straw, mushroom compost, food waste compost, protein, and starch. *S*. *piscinae* was isolated from the bottom of the southern fishpond. The colony is blue and performs aerobic growth. The aerial hyphae can produce non-moving short spore chains. It has high salt tolerance and is a salt-tolerant strain. More specialised physiological characteristics can produce special metabolites. It belongs to a special environment strain and is worthy of further investigation and study of its metabolites. However, investigation on the secondary metabolite of *Saccharomonospora* sp. is still very limited.

Nitric oxide (NO) is a mediator in the inflammatory response involved in host defense. In our effort to search for structurally interesting and bioactive natural products from microbes, we had isolated and identified over 300 microbial strains from Formosan plants and special environment research materials, and the crude EtOAc and BuOH extracts from those were screened for their inhibitory activity on lipopolysaccharide (LPS)-induced nitric oxide (NO) release production in RAW 264.7 murine macrophages. 

In the course of our search for potential diverse secondary metabolites from natural microbial sources, and to further understand the minor metabolites of the genus *Saccharomonospora*, we examined the EtOAc extract of *S*. *piscinae*, which showed rich metabolites according to the fingerprint analysis and inhibitory activity on LPS-induced NO release in RAW 264.7 murine macrophages, as determined by our primary screening (approximately 95% inhibition at a concentration of 10 μg/mL). Investigation of the bioactive metabolites of the active EtOAc extract from the microbe *S*. *piscinae*, fermented by solid rice medium, was investigated. The metabolites investigation guided by the HPLC profile analysis and 1D NMR spectra prescreening led to the isolation of three new metabolites, saccharpiscinols A–C (**1**–**3**, respectively), and three metabolites isolated for the first time from nature sources, (2*S*)-5,7,3′,4′-tetrahydroxy-6,8-dimethylflavanone (**4**), methyl-4-hydroxy-2-methoxy-6-methylbenzoate (**5**), and (±)-7-acetyl-4,8-dihydroxy-6-methyl-1-tetralone (**6**).The structures of these isolates were established by means of spectral experiments. The isolation, structural elucidation, and inhibitory effects of some isolates on nitric oxide (NO) production by RAW 264.7 macrophages are described herein.

## 2. Results

### 2.1. Structure Elucidation of Compounds

**Figure 1:**
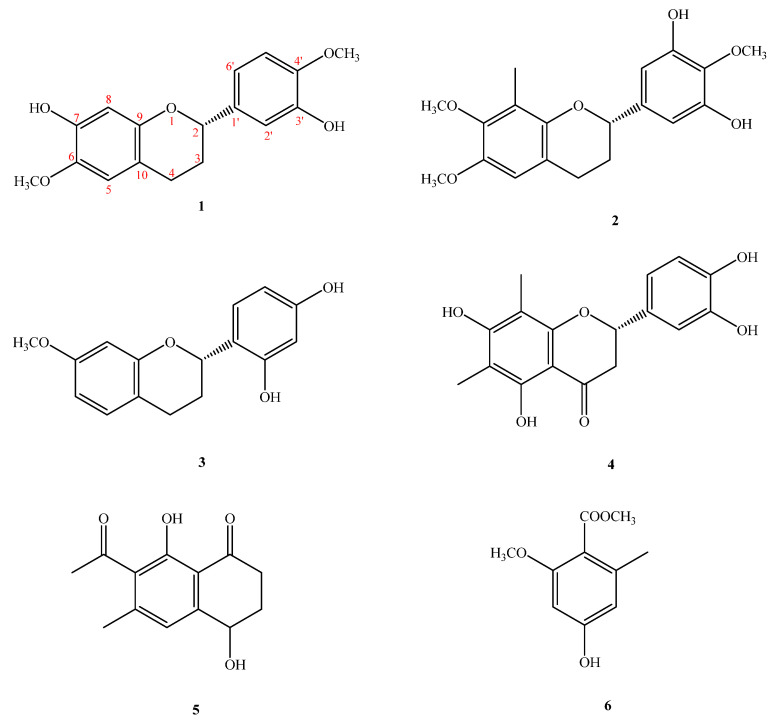
Compounds **1**–**6**, isolated from *Saccharomonospora piscinae* BCRC 16893.

**Figure 2:**
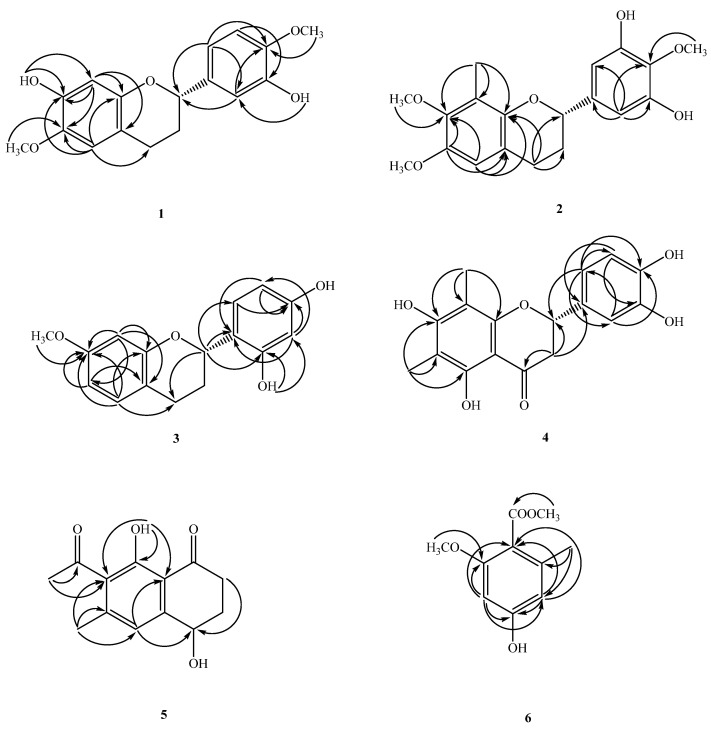
Key HMBC (→) correlations of **1**–**6**.

**Figure 3:**
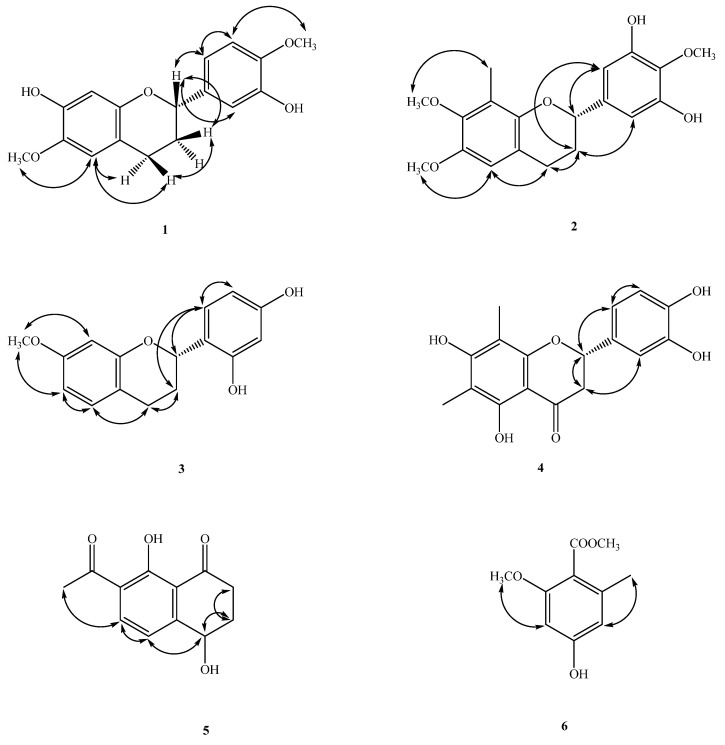
Major NOESY (↔) contacts of **1**–**6**.

Compound **1** was obtained as oil (Figure 1). The molecular formula, C_17_H_18_O_5_, was determined by electron impact (EI) ([M]^+^, *m*/*z* 302) and high-resolution (HR) mass spectrometry. The UV absorptions at 230 and 283 nm suggested the presence of a flavonoid skeleton [5]. The IR spectrum showed a broad band of OH absorption at 3340 cm^−1^ and aromatic absorptions at 1609 and 1510 cm^−1^. The ^1^H NMR data of **1** showed the resonances of a group of ABX aromatic protons [δ_H_ 6.97 (d, *J* = 2.0 Hz, H-2′), 6.89 (dd, *J* = 8.3, 2.0 Hz, H-6′), 6.82 (d, *J* = 8.3 Hz, H-5′)], two aromatic singlet protons at δ_H_ 6.52 (s, H-5) and 6.49 (s, H-8), four mutually-coupling aliphatic protons [δ_H_ 2.85 (ddd, *J* = 16.0, 11.0, 6.0 Hz, H-4a), 2.66 (ddd, *J* = 16.0, 5.0, 3.6 Hz, H-4b), 2.12(m, H-3a), 2.04 (m, H-3b)] in ring C, two methoxyl signals at δ_H_ 3.81, 3.87 (each 3H, s, OMe-6 and 4′), and two active hydrogens at δ_H_ 5.60 (1H, brs) and 5.74 (1H, brs). The ^1^H-^1^H COSY experiment of **1** revealed two spin systems of H-2/CH_2–_3/CH_2–_4 and H-5′/H-6′, which further confirmed the presence of resonances attributed to aliphatic protons belonging to the C ring, and aromatic resonances attributed to protons belonging to the B rings of flavan moieties. The ^13^C NMR data (see physical data) showed 17 carbon resonances, comprising 12 olefinic carbons, two methylene carbons (δ_C_ 24.7 and 30.0), two methoxyl carbons (δ_C_ 56.1 and 56.5), and an oxygenated methane carbon (δ_C_ 77.2). Analysis of the ^1^H and ^13^C NMR data of **1** (see physical data) exhibited a very close structural resemblance to (2*S*)-7-hydroxy-6-methoxyflavan [6], except for the replacement of the H-4′ and H-3′ [δ_H_ 7.2–7.4 (2H, m, H-3′ and H-4′) and 128.4 (C-3′), 127.7 (C-4′)] groups in (2*S*)-7-hydroxy-6-methoxyflavan by a methoxy group [δ_H_ 3.87 (3H, s)] at C-4′ and an OH group [δ_H_ 5.60 (1H, brs)] at C-3′ in **1**. In the HMBC experiment, the cross-peaks from OH-7 to C-7/C-8, from OH-3′ to C-2′, from the C-6-methyoxyl protons to C-6, and from OMe-4′ to C-4′, placed the methoxyl groups at C-6 and 4′, and the hydroxy groups at C-7 and 3′, respectively (Figure 2). The absolute configuration of the stereogenic center C-2 in **1** was corroborated by optical activity measurement. Compound **1** exhibited a negative optical-rotation value ([α${]}_{D}^{25}$–24 (*c* = 0.02, CHCl_3_)), indicating (*S*)-configuration at C-2 in comparison with the reported data of natural flavan analogues [5,6,7,8,9]. The above assignments were further confirmed by a NOESY experiment (Figure 3). Based on the above data, it can be determined that compound **1** is (2*S*)-7,3′-dihydroxy-6,4′-dimethoxyflavan. 

Compound **2** was isolated as oil ([α${]}_{D}^{25}$–15 (*c* = 0.02, CHCl_3_)). The molecular formula of C_18_H_20_O_5_ was established by HR and EI-mass spectrometry ([M]^+^, *m*/*z* 316). UV absorption at 231 and 278 nm indicated the presence of a flavan moiety [4]. The IR spectrum exhibited a hydroxyl absorption at 3423 cm^−1^ and an aromatic nucleus (1600, 1500 cm^−1^). Detailed analysis of the 1D- and 2D-NMR spectra revealed that **2** was a flavonoid derivative [5], very similar to that of (−)-4′-hydroxy-7-methoxy-8-methylflavan [10], except that a 2-methoxybenzene-1,3-diol moiety [(δ_H_ 3.95 (3H, s, OCH_3–_4′), 6.57(2H, s, H-2′, 6′); δ_C_ 61.1 (OMe-4′), 105.5 (C-2′ and 6′), 133.7 (C-1′), 139.3 (C-4′), 148.8 (C-3′)] in **2** replaced a 4-phenol moiety [δ_H_ 7.32 (2H, d, *J* = 8.8 Hz, C-2′, 6′) and 6.94 (2H, d, *J* = 8.8 Hz, C-3′, 5′) and δ_C_ 155.2 (C-4′), 129.9 (C-1′), 127.6 (C-2′, 6′), 115.3 (C-3′, 5′)] in the C-2 position in (−)-4′-hydroxy-7-methoxy-8-methylflavan [10]. The location of one of the methoxy groups at C-7 and the Me group at C-8 were inferred from the HMBC correlations of the methoxy protons with C-7, H-5 with C-7, and H-6 with C-8 (Figure 2). The remaining methoxy group at C-4′, and two hydroxyl groups at C-3′ and 5′ were deduced from the HMBC correlations from OCH_3–_4′ to C-4′, and from the aromatic protons (H-2′) to C-2, 1′, 3′, 4′, and 6′ (Figure 2). The levorotatory optical activity ([α${]}_{D}^{24}$–15 (*c* = 0.02, CHCl_3_)) once again indicated the stereochemistry of C-2 as 2*S* [5,6,7,8]. Taken together, the structure of compound **2** was assigned as (2*S*)-3′,5′-dihydroxy-7,4′-dimethoxy-8-methylflavan.

Compound **3** was isolated as optically active oil with [α${]}_{D}^{25}$–21 (*c* = 0.01, CHCl_3_). The EI-mass spectrum afforded the positive ion at *m*/*z* 272 [M]^+^, implying a molecular formula of C_16_H_16_O_4_. This was confirmed by the HREI-mass spectrum ([M]^+^ found 272.1047, calcd 272.1049). The UV absorptions (232 and 283 nm) were similar to those of (−)-4′-hydroxy-7-methoxy-8-methylflavan [10] and were characteristic of the flavan skeleton. The IR spectrum showed absorptions due to a hydroxyl group (3428 cm^−1^) and an aromatic ring (1626 and 1457 cm^−1^). The ^1^H NMR data of **3** (see physical data) contained the following resonances: a methoxy signal at δ_H_ 3.73 (s), two ABX-spin-coupling system with δ_H_ 6.44 (d, *J* = 2.4 Hz, H-8), 6.50 (dd, *J* = 8.4, 2.4 Hz, H-6), 6.98 (d, *J* = 8.4 Hz, H-5), 6.37 (dd, *J* = 8.4, 2.4 Hz, H-5′), 6.97 (d, *J* = 8.4 Hz, H-6′), 6.40 (d, *J* = 2.4 Hz, H-3′), one oxymethine at δ_H_ 5.11 (dd, *J* = 10.0, 2.4 Hz), two methylenes at δ_H_ 2.14–2.28 (m, CH_2–_3), 2.93 (ddd, *J* = 16.0, 12.0, 7.2 Hz, H-4β), 2.79 (ddd, *J* = 16.0, 5.2, 2.4 Hz, H-4α), and two active hydrogens at δ_H_ 7.02 (1H, brs) and 5.25 (1H, brs). The NMR data of **3** were found to be similar to those of (2*S*)-2′,4′-dihydroxy-7-methoxy-8-methylflavan [11], and the apparent difference was the replacement of an aromatic singlet proton (δ_H_ 6.44) at C-8 in **3** by a Me singlet at C-8 in (2*S*)-2′,4′-dihydroxy-7-methoxy-8-methylflavan. This was supported by the HMBC correlations between H-8 (δ_H_ 6.44) and C-6 (δ_C_ 10.3), C-7 (δ_C_ 158.4), C-9 (δ_C_ 153.8), and C-10 (δ_C_ 113.8), and the NOESY correlations between H-8 (δ_H_ 6.44) and OCH_3–_7 (δ_H_ 3.73). With the aid of 1D and 2D NMR experiments, all the ^1^H and ^13^C NMR data were completely assigned. In addition, the negative [α${]}_{D}^{25}$–21 (*c* = 0.01, CHCl_3_) value indicated that C-2 was in *S* configuration, which was supported by comparison of the [α]_D_ value of **3** with (2*S*)-2′,4′-dihydroxy-7-methoxy-8-methylflavan [5,6,7,8,9,11]. Therefore, the structure of **3** was established as (2*S*)-2′,4′-dihydroxy-7-methoxyflavan.

Compound **4** was obtained as yellow syrup ([α${]}_{D}^{25}$ –22 (*c* = 0.014, CHCl_3_)). The molecular formula was determined as C_17_H_16_O_6_ on the basis of the [M]^+^ peak at *m*/*z* 316.0945 (calcd. 316.0947 for C_17_H_16_O_6_) in its HR-EI-MS. The UV absorptions (λ_max_ 231, 291 nm) confirmed the presence of a flavonoid nucleus [5]. The IR spectrum showed typical absorption bands of hydroxyl and carbonyl groups at 3394 and 1721 cm^−1^, respectively. The ^1^H and ^13^C NMR spectra of **4** are quite similar to those of (2*S*)-farrerol [12], which suggested that compound **4** has a flavanone structure. Comparing the ^1^H and ^13^C NMR spectra of compound **4** with those of (2*S*)-farrerol, it was presumed that an aromatic proton at C-3′ of (2*S*)-farrerol was substituted with an OH moiety in **4,** suggested from an ABX pattern system [δ_H_ 6.79 (brs, H-2′ and 5′), 6.93 (1H, s, H-6′); δ_C_ 114.6 (C-6′), 116.2 (C-5′), 119.0 (C-2′), 146.5 (C-3′), 146.7 (C-4′)] in the B ring which were confirmed by HMBC correlations (Figure 2). Based on these data, the structure of compound **4** was defined as shown, and the compound was named (2*S*)-5,7,3′,4′-tetrahydroxy-6,8-dimethylflavanone. This is the first time that compound **4** has been isolated from a natural source, although it had previously been synthesized [13].

Compound **5** was identified by comparison with literature data of 7-acetyl-4*R*,8-dihydroxy-6-methyl-1-tetralone [13] but showed a zero optical activity with [α${]}_{D}^{24}$±0 (*c* = 0.01, CHCl_3_). Isolate **6** showed zero optical activity with [α${]}_{D}^{25}$±0 (*c* = 0.01, CHCl_3_), and by reference to (+)-7-acetyl-4*R*,8-dihydroxy-6-methyl-1-tetralone [13], the configuration of 10 at C-4 was proposed as being in the (R/S)-form. Thus, the isolation of (±)-7-acetyl-4*R*,8-dihydroxy-6-methyl-1-tetralone (**5**) as a natural product is also reported for the first time. Compound **5** was identified by comparison with literature data of 7-acetyl-4*R*,8-dihydroxy-6-methyl-1-tetralone [14] but showed a zero optical activity with [α${]}_{D}^{25}$±0 (*c* = 0.01, CHCl_3_). Isolate **5** showed zero optical activity with [α${]}_{D}^{25}$±0 (*c* = 0.01, CHCl_3_), and by reference to (+)-7-acetyl-4*R*,8-dihydroxy-6-methyl-1-tetralone [13], the configuration of 10 at C-4 was proposed as being in the (*R*/*S*)-form. Thus, the isolation of (±)-7-acetyl-4*R*,8-dihydroxy-6-methyl-1-tetralone (**5**) as a natural product is also reported for the first time.

Compound **6** was obtained as gum. Its molecular formula, C_10_H_12_O_4_, was deduced from a [M]^+^ peak at *m*/*z* 196.0733 in its HREIMS spectrum (calcd. 196.0736 for C_10_H_12_O_4_). There are 10 carbon signals in the ^13^C NMR spectrum, including one ester C=O at δ_C_ 168.0, one methyl group at δ_C_ 19.7, two methoxyls at δ_C_ 51.7 and 55.9, and six aromatic carbon atoms with one deshielded signal at δ_C_ 159.6. The ^1^H and ^13^C NMR spectra of **5** are quite similar to those of methyl-2,4-dihydroxy-6-methyl benzoate, which suggested that compound **5** has a benzenoid structure. Comparing the ^1^H and ^13^C NMR spectra of compound **5** with those of the similar analogue, methyl-2,4-dihydroxy-6-methyl benzoate, it was presumed that a hydroxyl group [δ_H_ 11.60 (1H, s, OH-2); δ_C_ 163.24 (C-2)] at C-2 of methyl-2,4-dihydroxy-6-methyl benzoate was substituted with a methoxy moiety in **8** from the signals at [δ_H_ 3.72 (3H, s, OMe-2); δ_C_ 55.9 (OMe-2)], which were confirmed by HMBC correlations. The HMBC correlations of OMe-2/C-2′ and Me-6/C-6, 1 and 5 determined the positions of methoxyl and methyl groups, which was confirmed by the correlations between OMe-2/H-3 and Me-6/H-5 in the NOESY spectrum (Figure 3). Finally, the NMR data were identical to those of methyl-4-hydroxy-2-methoxy-6-methylbenzoate, which has been obtained synthetically [15]. This is the first time **6** has been isolated from a nature source.

#### Analysis EtOAc Crude Extracts of Compounds by HPLC 

**Figure 4:**
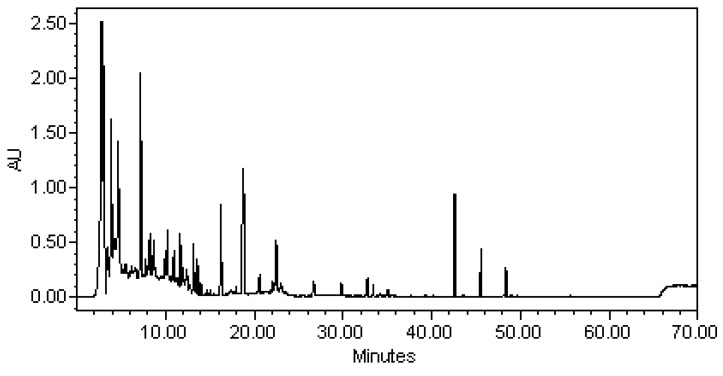
HPLC fingerprints of EtOAc extracts from the rice fermentation of the actinobacteria, *Saccharomonospora piscinae*, λ = 237 nm.

The EtOAc extracts from the rice fermentation of the actinobacteria, *S*. *piscinae* was analyzed by HPLC and detection by UV spectroscopy (waters 600 pump and 996 photodiode array detector) on an Inertsil ODS-3 column, 5 μm, 250 mm × 4.6 mm i.d. (GL Sciences, Tokyo, Japan) using gradient of (A) water containing 0.1% phosphoric acid and (B) acetonitrile: 0–50 min, 90–50% A; 50–80 min, 50% A-100% B; flow 1 mL/min. Chromatograms were recorded at wavelength 237 nm. (Figure 4).

### 2.2. Biological Studies

Due to the small quantity of isolated compounds (**5** and **6**), we evaluated the inhibitory effects of saccharpiscinols A–C (**1**–**3**, respectively) and (2*S*)-5,7,3′,4′-tetrahydroxy-6,8-dimethylflavanone (**4**) on the production of NO induced by LPS (Table 1). They showed potent inhibition with IC_50_ values between 12.5 to 21.8 μM against lipopolysaccharide (LPS)-induced nitric oxide (NO) generation. The high cell viability (>80%) indicated that the inhibitory activity of LPS-induced nitrite production by compounds **1** and **2** (IC_50_ value: 12.5 μM and 18.0 μM) did not result from its cytotoxicity. Compounds **3** and **4** (IC_50_ value: 21.8 μM and 20.0 μM) also showed inhibition of the NO production of macrophages, but the low cell viability (<80%) suggested the possibility of cytotoxicity.

## 3. Materials and Methods

### 3.1. General Experimental Procedures

TLC: silica gel 60 F_254_ precoated plates (Merck). Column chromatography (CC): silica gel 60 (70–230 or 230–400 mesh, Merck) and Spherical C18 100A Reversed Phase Silica Gel (RP-18) (particle size: 20–40 μm) (Silicycle). HPLC: Spherical C18 column (250 × 10 mm, 5μm) (Waters); LDC-Analytical-III apparatus. UV Spectra: Jasco UV-240 spectrophotometer; λ_max_ (log ε) in nm. Optical rotation: Jasco DIP-370 polarimeter; in CHCl_3_. IR Spectra: Perkin-Elmer-2000 FT-IR spectrophotometer; ν in cm^−1^. ^1^H-, ^13^C- and 2D-NMR spectra: Varian-Mercury-400 and Varian-Unity-Plus-400 spectrometers; *δ* in ppm relative to Me_4_Si, *J* in Hz. ESI and HRESIMS: Bruker APEX-II mass spectrometer; in *m*/*z*.

#### 3.1.1. Microorganism

*Saccharomonospora piscinae* (06168H-1^T^) was used throughout this study, and deposited at the Bioresource Collection and Research Center (BCRC), Food Industry Research and Development Institute (FIRDI). This actinobacteria was identified by M. T.

#### 3.1.2. Cultivation and Preparation of the Fungal Strain

The actinobacteria, *Saccharomonospora piscinae*, (06168H-1^T^), was isolated from dried fishpond sediment from the Kouhu township, in the south of Taiwan. The sample of fishpond sediment was dried at room temperature for 7 days. The sample suspension (100 µL) was plated on modified Sabouraud glucose agar (SGA; 7.5 g casamino acid, 10.0 g yeast extract, 20.0 g MgSO4·7H_2_O, 3.0 g trisodium citrate·2H_2_O, 2.0 g KCl, 34.0 g NaCl, 10.0 µg Fe^2+^, 18.0 g agar, 1.0 l distilled water, pH adjusted to 7.4) and incubated at 30 °C for 4 weeks. The spores or mycelia suspension were harvested with 20% (*v*/*v*) glycerol and stored at −20 °C.

The actinobacteria, *Saccharomonospora piscinae*, (06168H-1^T^), was maintained on Sabouraud glucose agar (SGA) and the strain was cultured on potato dextrose agar slants at 30 °C for 7 days, and then the spores were harvested by sterile water. The spores (5 × 10^5^) were seeded into 500 mL shake flasks containing 50 mL CM+YM (Sabouraud glucose agar, SGA; 7.5 g casamino acid, 10.0 g yeast extract, 20.0 g MgSO4·7H_2_O, 3.0 g trisodium citrate·2H_2_O, 2.0 g KCl, 34.0 g NaCl, 10.0 µg Fe^2+^, 18.0 g agar, 1.0 L distilled water, pH adjusted to 7.4) and cultivated with shaking (150 rpm) at 30 °C for 5 days. After the mycelium enrichment step, an inoculum mixing 100 mL mycelium broth and 100 mL CM+YM medium was inoculated into plastic boxes (25 cm × 30 cm) containing 1.3 kg sterile rice and cultivated at 30 °C for producing rice, and the abovementioned CM+YM medium was added to maintain the growth. After 21 days of cultivation, the rice was harvested, and used as a sample for further extraction.

#### 3.1.3. Isolation and Characterization of Secondary Metabolites

The rice fermentation was extracted five times with an equal volume of EtOAc. The combined EtOAc layers were evaporated to dryness under reduced pressure to give an EtOAc extract (12 g) which was subjected to silica gel column chromatography (CC) (petroleum ether-EtOAc *v*/*v*, gradient; from 10:1 to 0:1 to generate seven fractions (Frs. 1–7). Fr. 2 was isolated by CC on silica gel eluted with hexane-EtOAc (from 5:1 to 1:1) to afford four subfractions (Fr. 2.1–2.4). Subfraction Fr. 2.3 was further purified by semi-preparative HPLC (CH_3_CN/H_2_O, 40:60 *v*/*v*) to obtain **6** (1.2 mg). Subfraction Fr. 2.4 was further separated by HPLC (CH_3_CN/H_2_O, 40:60 *v*/*v*) to obtain **5** (1.51 mg). Compound **3** (10.4 mg) was preferentially precipitated from Fr. 3. Fr. 5 was separated into 13 subfractions (Fr. 5–1—Fr. 5–13) by silica gel column chromatography eluted with a step gradient of petroleum ether–EtOAc (from 1:0 to 0:1, *v*/*v*). Fr. 5–2 was subjected to analytical RP-HPLC (85% MeOH–H_2_O) to obtain **4** (3.2 mg). Fr. 5–5 was also purified by analytical RP-HPLC (77.5% MeOH–H_2_O) to afford compounds **1** (3.2 mg) and **2** (10.4 mg).

Saccharpiscinol A (**1**): oil; [α]25 D −24 (*c* 0.01, CHCl_3_); UV (MeOH): 230 (4.14), 283.0 (3.85) nm; IR (Neat): 3400 (OH), 1609, 1510 (aromatic C=C) cm^−1^; ^1^H NMR (400 MHz, CDCl_3_): δ_H_ 6.97 (1H, d, *J* = 2.0 Hz, H-2′), 6.89 (1H, dd, *J* = 8.3, 2.0 Hz, H-6′), 6.82 (1H, d, *J* = 8.3 Hz, H-5′), 6.52 (1H, s, H-5), 6.49 (1H, s, H-8), 5.60 (1H, br.s, OH-3′), 5.47 (1H, br.s, OH-7), 4.88 (1H, dd, *J* = 10.0, 2.4 Hz, H-2), 3.87 (3H, s, OCH_3–_4′), 3.81 (3H, s, OCH_3–_6), 2.85 (1H, ddd, *J* = 16.0, 11.0, 6.0 Hz, H-2β), 2.66 (1H, ddd, *J* = 16.0, 5.0, 3.6 Hz, H-2α), 2.12 (1H, m, H-3β), 2.04 (1H, m, H-3α); ^13^C NMR (100 MHz, CDCl_3_): δ_c_ 149.2 (C-9), 146.2 (C-4′), 145.6 (C-3′), 144.8 (C-7), 140.8 (C-6), 134.7 (C-1′), 112.2 (C-2′), 112.5 (C-10), 117.8 (C-6′), 111.4 (C-5), 110.5 (C-5′), 103.5 (C-8), 77.2 (C-2), 56.5 (OMe-6), 56.1 (OMe-6), 24.7 (C-4), 30.0 (C-3); HREIMS *m*/*z* 302.1153 [M]^+^ (calcd. for C_17_H_18_O_5_, 302.1154).

Saccharpiscinol B (**2**): oil; [α]25 D = −15 (*c* 0.01, CHCl_3_); UV (MeOH): 230 (4.14), 283.0 (3.85) nm; IR (Neat): 3400 (OH), 1609, 1510 (aromatic C=C) cm^−1^; ^1^H NMR (400 MHz, CDCl_3_): δ_H_ 6.84 (1H, d, *J* = 8.3 Hz, H-5), 6.57 (1H, s, H-6′), 6.43 (1H, d, *J* = 8.3 Hz, H-6), 4.92 (1H, dd, *J* = 10.0, 2.1 Hz, H-2), 3.95 (3H, s, OCH_3–_4′), 3.85 (3H, s, OMe-7), 2.87 (1H, ddd, *J* = 16.0, 10.5, 8.3 Hz, H-4β), 2.70 (1H, ddd, *J* = 16.0, 8.8, 4.5 Hz, H-4α), 2.15 (1H, m, H-3β), 2.10 (3H, s, Me-8), 1.93 (1H, m, H-3α); ^13^C NMR (100 MHz, CDCl_3_): δ_c_ 156.7 (C-7), 153.2 (C-9), 148.8 (C-3′ and 5′), 139.3 (C-4′), 133.7 (C-1′), 126.1 (C-5), 114.2 (C-10), 113.8 (C-8), 105.5 (C-2′ and 6′), 103.1 (C-6), 77.2 (C-2), 61.1 (OMe-4′), 55.8 (OMe-7), 30.0 (C-3), 24.6 (C-4), 8.7 (Me-8); HREIMS *m*/*z* 316.1310 [M]^+^ (calcd. for C_18_H_20_O_5_, 316.1308).

Saccharpiscinol C (**3**): oil; [α]25 D = −21.0 (*c* 0.01, CHCl_3_); UV (MeOH): 232 (4.08), 283 (3.69) nm; IR (Neat): 3428 (OH), 1626 (aromatic C=C) cm^−1^; ^1^H NMR (400 MHz, CDCl_3_): δ_H_ 7.02 (1H. br.s, OH-2′), 6.98 (1H, d, *J* = 8.4 Hz, H-5), 6.97 (1H, d, *J* = 8.4 Hz, H-6′), 6.50 (1H, dd, *J* = 8.4, 2.4 Hz, H-6), 6.44 (1H, d, *J* = 2.4 Hz, H-8), 6.40 (1H, d, *J* = 2.4 Hz, H-3′), 6.37 (1H, dd, *J* = 8.4, 2.4 Hz, H-5′), 5.25 (1H, br.s, OH-2′), 5.11 (1H, dd, *J* = 10.0, 2.4 Hz, H-2), 3.73 (3H, s, OMe-7), 2.93 (ddd, *J* = 16.0, 12.0, 7.2 Hz, H-4β), 2.79 (1H, ddd, *J* = 16.0, 5.2, 2.4 Hz, H-4α), 2.14–2.28 (2H, m, H-3α and β); ^13^C NMR (100 MHz, CDCl_3_): δ_c_ 158.4 (C-7), 156.1 (C-2′), 155.6 (C-4′), 153.8 (C-9), 129.9 (C-5), 127.7 (C-6′), 108.3 (C-6), 117.7 (C-1′), 113.8 (C-10), 107.2 (C-5′), 104.1 (C-3′), 101.6 (C-8), 78.3 (C-2), 55.5 (OMe-7), 28.5 (C-3), 24.7 (C-4); EIMS (70 eV) *m*/*z* (%): 272 ([M]^+^, 100), 150 (42), 137 (90), 101 (20); HREIMS *m*/*z* 272.1047 [M]^+^ (calcd. for C_16_H_16_O_4_, 272.1049).

The compound, (2*S*)-5,7,3′,4′-Tetrahydroxy-6,8-dimethylflavanone (4): oil; [α]25 D = −22 (*c* 0.01, CHCl_3_); UV (MeOH): 231 (4.44), 291 (4.35) nm; IR (Neat): 3394 (OH), 1721 (conjugated C=O), 1619, 1453 (aromatic C=C) cm^−1^; ^1^H NMR (400 MHz, CDCl_3_): δ_H_ 6.93(1H, s, H-6′), 6.80 (1H, br.s, H-5′), 6.79 (1H, br.s, H-2′), 5.24 (1H, dd, *J* = 12.5, 3.0 Hz, H-2), 3.02 (1H, dd, *J* = 16.9, 12.5 Hz, H-3α), 2.71 (1H, dd, *J*= 16.9, 3.0 Hz, H-3β), 2.00 (3H, s, Me-8), 1.99 (3H, s, Me-6); ^13^C NMR (100 MHz, CDCl_3_): δ_c_ 198.4 (C-4), 164.1 (C-7), 160.2 (C-5), 159.3 (C-9), 146.7 (C-4′), 146.5 (C-3′), 132.2 (C-1′), 119.0 (C-2′), 116.2 (C-5′), 114.6 (C-6′), 104.7 (C-10), 104.1 (C-6), 103.3 (C-8), 80.0 (C-2), 44.2 (C-3), 8.2 (C-6), 7.4 (Me-8); EIMS (70 eV) *m*/*z* (%): 316 [M]^+^(100), 286 (12), 255 (10), 207 (13), 194 (20), 181 (68), 152 (52), 136 (17), 55 (10); HREIMS *m*/*z* 316.0945 [M]^+^ (calcd. for C_17_H_16_O_6_, 316.0947).

The compound, (±)-7-Acetyl-4,8-dihydroxy-6-methyl-1-tetralone (**5**): oil; [α${]}_{D}^{25}$±0 (*c* 0.01, CHCl_3_); UV (MeOH): 210 (4.56), 238.0 (4.61), 272.0 (4.40), 336.0 (4.17) nm; IR (Neat): 3428 (OH), 1796 (C=O), 1626, 1552 (aromatic C=C) cm^−1^; ^1^H NMR (400 MHz, CDCl_3_): δ_H_ 12.78 (1H, s, OH-8), 6.85 (1H, s, H-5), 4.79 (1H, dd, *J* = 7.9, 3.7 Hz, H-4), 2.88 (1H, ddd, *J* = 17.8, 7.3, 4.6 Hz, H-2eq), 2.57 (1H, ddd, *J* = 17.8, 9.1, 4.7 Hz, H-2ax), 2.50 (3H, s, CH_3–_11), 2.28 (1H, m, H-3eq), 2.23 (3H, s, CH_3–_6), 2.10 (1H, m, H-3ax); ^13^C NMR (100 MHz, CDCl_3_): δ_c_ 204.2 (C-11), 203.9 (C-1), 160.0 (C-8), 146.9 (C-10), 145.3 (C-6), 129.7 (C-7), 119.3 (C-5), 113.2 (C-9), 67.3 (C-4), 34.7 (C-2), 32.0 (CH_3–_11), 31.2 (C-3), 20.3 (CH_3–_6); EIMS (70 eV) *m*/*z* (%): 234 ([M]^+^, 35), 219 (100), 201 (40), 145 (20), 77 (18), 55 (20); HREIMS *m*/*z* 234.0889 [M]^+^ (calcd. for C_13_H_14_O_4_, 234.0892).

Methyl-4-hydroxy-2-methoxy-6-methylbenzoate (**6**): gum; UV (MeOH): 221.0 (4.15), 253.0 (3.69), 281.0 (3.56) nm; IR (Neat): 3370 (OH), 1701 (ester C=O), 1604, 1454 (aromatic C=C) cm^−1^; ^1^H NMR (400 MHz, CDCl_3_): δ_H_ 8.57 (1H, br.s, OH-4), 6.34 (1H, d, *J* = 1.8 Hz, H-3) 6.29 (1H, d, *J* = 1.8 Hz, H-5), 3.76 (3H, s, OCH_3–_7), 3.72 (3H, s, OCH_3–_2), 2.16 (3H, s, CH_3–_6); ^13^C NMR (100 MHz, CDCl_3_): δ_c_ 168.0 (C-7), 159.2 (C-4), 158.4 (C-2), 137.8 (C-6), 116.0 (C-1), 109.1 (C-5), 97.1 (C-3), 55.9 (OCH_3–_2), 51.7 (OCH_3–_7), 19.7 (CH_3–_6); EIMS (70 eV) *m*/*z* (%): 196 ([M]^+^, 32), 165 (100), 122 (10); HREIMS *m*/*z* 196.0733 [M]^+^ (calcd. for C_10_H_12_O_4_, 196.0736).

#### 3.1.4. Determination of NO Production and Cell Viability Assay

Mouse macrophage cell line (RAW 264.7) was obtained from the Bioresource Collection and Research Center (BCRC 60001) and cultured at 37 °C in Dulbecco’s Modified Eagle’s Medium (DMEM) supplemented with 10% fetal bovine serum (Gibco), 4.5 g/L glucose, 4 mM glutamine, penicillin (100 units/mL), and streptomycin (100 μg/mL) in a humidified atmosphere in a 5% CO_2_ incubator. The cells were treated with 10, 25, and 50 μM of natural products in the presence of 1 μg/mL LPS (lipopolysaccharide, Sigma-Aldrich) for 20 h. The concentration of NO in culture supernatants was determined as nitrite, a major stable product of NO, by a Griess reagent assay [16], and cell viabilities were determined using the MTT assay as described previously [17].

## 4. Conclusions

Under the support of the Ministry of Economic Affairs, the Bioresource Collection and Research Center at the Food Industry Research and Development Institute has been dedicated to the research work on the collection, separation, and preservation of bio-resource research in the past few years, and has constructed a complete indigenous strains resource bank in Taiwan. The applicant analyzed the active constituents from it, and obtained more than sixty active new compounds isolated from red yeast rice, endophytes, actinobacteria, and mushrooms, among which many new compounds have anti-cancer and anti-inflammatory effects [18,19,20,21,22,23].

Actinobacteria have the ability to produce a variety of physiologically active products, so they play a very important role in the food and pharmaceutical industries. Over the years, our team has also separated and collected actinomycetes resources from all over Taiwan and various environments. In addition to common *Streptomyces*, there are also many rare species of actinobacteria, and there are many new species. Based on the concept of “new species and new compounds”, it is expected that special compounds can be found from these new strains. In recent years, studies have also found that these new species of actinobacteria can produce many active secondary metabolites. In order to further explore the efficacy of different strains of actinobacteria and expand the application range of actinomycetes, therefore, this project uses one new species of actinobacteria that has not been studied in the past. This species is cultured, extracted, purified and identified with high-level and highly active anti-inflammatory compounds with solid rice, in order to improve the research of actinobacteria at the country level, and develop health products related to actinobacteria. In summary, we have isolated and characterized three undescribed flavan derivatives, saccharpiscinols A–C, from an actinobacteria strain *Saccharomonospora piscinae* that was isolated from dried fishpond sediment from the Kouhu township, in the south of Taiwan. The absolute configurations of saccharpiscinols A–C were determined by comparing their optical activities with related derivatives. Saccharpiscinols A and B showed inhibitory activities against LPS-induced NO production. The discovery of flavan derivatives from actinobacteria pointed toward the potential use of endophytic or associated *Saccharomonospora piscinae* as alternative producers of flavan derivatives. The current results may encourage further investigations on the chemistry and bioactivity of flavan metabolites. These results also suggest that *Saccharomonospora* has distinct and diverse metabolites that arise under different fermentation conditions and soil-derived collections. It may therefore be possible to find more new bioactive natural products by searching *Saccharomonospora* species under special eco-environments. For the sake of better understanding the distribution of flavan acid analogs, the actinobacteria of the tilted research material and other special strains are worth examining for the presence of these secondary metabolites.

## Figures and Tables

**Table 1:** Effects of compounds (**1**–**4**) on LPS-induced NO production and cell viability in RAW 264.7 macrophages.

Compound	IC_50_	Cell Viability
	Nitrite (μM)	(% Control)
**1**	12.5 ± 0.2 *	91.1 ± 4.2
**2**	18.0 ± 0.3 *	82.2 ± 3.2 ^#^
**3**	21.8 ± 0.5 *	76.3 ± 5.4 ^#^
**4**	20.0 ± 1.5 *	68.2 ± 3.3 ^#^
Quercetin	35.45 ± 3.78 *	95.9 ± 1.3

The RAW 264.7 cells were incubated with compounds in the presence of LPS (1 μg/mL). The medium was harvested 24 h later and assayed for nitrite production. NO release was measured by using the Griess reagent. The cell viability was evaluated with the MTT assay. The results are presented as a percentage of the control value obtained from non-treated cells. Values are expressed as mean ± SD of three individual experiments, performed in triplicate. * Statistically significant difference compared to LPS-activated cells (*p* < 0.05). ^#^ Statistically significant difference compared to untreated cells (*p* < 0.05).

## Data Availability

The data presented in this study are available in the article and Supplementary Materials.

## Supplementary Materials

The following are available online, Figures S1~S7 1D, 2D NMR, and EIMS spectra for 1, Figures S8~S14 1D, 2D NMR, and EIMS spectra for 2, Figures S15~S21 1D, 2D NMR, and EIMS spectra for 3, Figures S22~S28 1D, 2D NMR, and EIMS spectra for 4, Figures S29~S35 1D, 2D NMR, and EIMS spectra for 5, Figures S36~S42 1D, 2D NMR, and EIMS spectra for 6.

## Sample Availability

Samples of the compounds are available from the authors.
