# Supplementary material for: Saccharpiscinols A–C: Flavans with Potential Anti-Inflammatory Activities from One Actinobacteria Saccharomonospora piscinae
Source: Molecules. 2021 Aug 13;26(16):4909. doi: 10.3390/molecules26164909 (PMC8398073; doi:10.3390/molecules26164909)
Supplement: Supplementary file 1 [file molecules-26-04909-s001.zip › molecules-1305768-supplementary.pdf]

Supplementary File

Saccharpiscinols A–C, Flavans with Potential inflammatory Activities from one Actinobacteria *Saccharomonospora piscinae*

Yung-Shun Su[1,2], Jih-Jung Chen[3,4], Ming-Jen Cheng[5*], Chee-Yin Chai,[1,6] Aij-Lie Kwan,[1,7,8*] Jheng-Cian Huang[9] and Yueh-Hsiung Kuo[4,9,10,11*]

1 Graduate Institute of Medicine, College of Medicine, Kaohsiung Medical University (KMU), Kaohsiung 807, Taiwan, e-mail: mariussu@gmail.com
2 Department of Dermatology, Kaohsiung Medical University Chung-Ho Memorial Hospital, Kaohsiung 807, Taiwan
3 Faculty of Pharmacy, School of Pharmaceutical Sciences, National Yang Ming Chiao Tung University (NYCU), Taipei 112, Taiwan, e-mail: jjungchen@nycu.edu.tw
4 Department of Medical Research, China Medical University Hospital, Taichung 404, Taiwan
5 Bioresource Collection and Research Center (BCRC), Food Industry Research and Development Institute (FIRDI), Hsinchu 300, Taiwan, e-mail: chengfirdi@gmail.com
6 Department of Pathology, Kaohsiung Medical University Chung-Ho Memorial Hospital, Kaohsiung 807, Taiwan, e-mail: ccjtsai@yahoo.com
7 Ph.D. Program in Environmental and Occupational Medicine, College of Medicine, Kaohsiung Medical University and National Health Research Institutes, Kaohsiung 807, Taiwan
8 Department of Neurosurgery, Kaohsiung Medical University Chung-Ho Memorial Hospital, Kaohsiung 807, Taiwan
9 Department of Chemistry, National Taiwan University, Taipei 106, Taiwan
10 Department of Biotechnology, Asia University, Taichung 413, Taiwan
11 Department of Chinese Pharmaceutical Sciences and Chinese Medicine Resources, College of Pharmacy, China Medical University, Taichung 404, Taiwan

*Correspondences: chengfirdi@gmail.com (M.-J.C.); aijliekwan@yahoo.com.tw (A.-L.K.); yhkuo800@gmail.com (Y.-H.K)

Contents

Figure S1. ^{1}H NMR spectrum of **1**........ 4
Figure S2. ^{13}C NMR spectrum of **1**........ 4
Figure S3. ^{1}H-^{1}H COSY spectrum of **1**........ 5
Figure S4. HMBC spectrum of **1**........ 5
Figure S5. NOESY spectrum of **1**........ 6
Figure S6. HSQC spectrum of **1**........ 6
Figure S7. EI-MS spectrum of **1**........ 7

Figure S8. ^{1}H NMR spectrum of **2**........ 8
Figure S9. ^{13}C NMR spectrum of **2**........ 8
Figure S10. ^{1}H-^{1}H COSY spectrum of **2**........ 9
Figure S11. HMBC spectrum of **2**........ 9
Figure S12. NOESY spectrum of **2**........ 10
Figure S13. HSQC spectrum of **2**........ 10
Figure S14. EI-MS spectrum of **2**........ 11

Figure S15. ^{1}H NMR spectrum of **3**........ 12
Figure S16. ^{13}C NMR spectrum of **3**........ 12
Figure S17. ^{1}H-^{1}H COSY spectrum of **3**........ 13
Figure S18. HMBC spectrum of **3**........ 13
Figure S19. NOESY spectrum of **3**........ 14
Figure S20. HSQC spectrum of **3**........ 14
Figure S21. EI-MS spectrum of **3**........ 15

Figure S22. ^{1}H NMR spectrum of **4**........ 16
Figure S23. ^{13}C NMR spectrum of **4**........ 16
Figure S24. ^{1}H-^{1}H COSY spectrum of **4**........ 17
Figure S25. HMBC spectrum of **4**........ 17
Figure S26. NOESY spectrum of **4**........ 18

Figure S27. HSQC spectrum of **4** 18

Figure S28. EI-MS spectrum of **4** 19

Figure S29. ^{1}H NMR spectrum of **5** 20

Figure S30. ^{13}C NMR spectrum of **5** 20

Figure S31. ^{1}H-^{1}H COSY spectrum of **5** 21

Figure S32. HMBC spectrum of **5** 21

Figure S33. NOESY spectrum of **5** 22

Figure S34. HSQC spectrum of **5** 22

Figure S35. EI-MS spectrum of **5** 23

Figure S36. ^{1}H NMR spectrum of **6** 24

Figure S37. ^{13}C NMR/DEPT spectra of **6** 24

Figure S38. ^{1}H-^{1}H COSY spectrum of **6** 25

Figure S39. HMBC spectrum of **6** 25

Figure S40. NOESY spectrum of **6** 26

Figure S41. HSQC spectrum of **6** 26

Figure S42. EI-MS spectrum of **6** 27

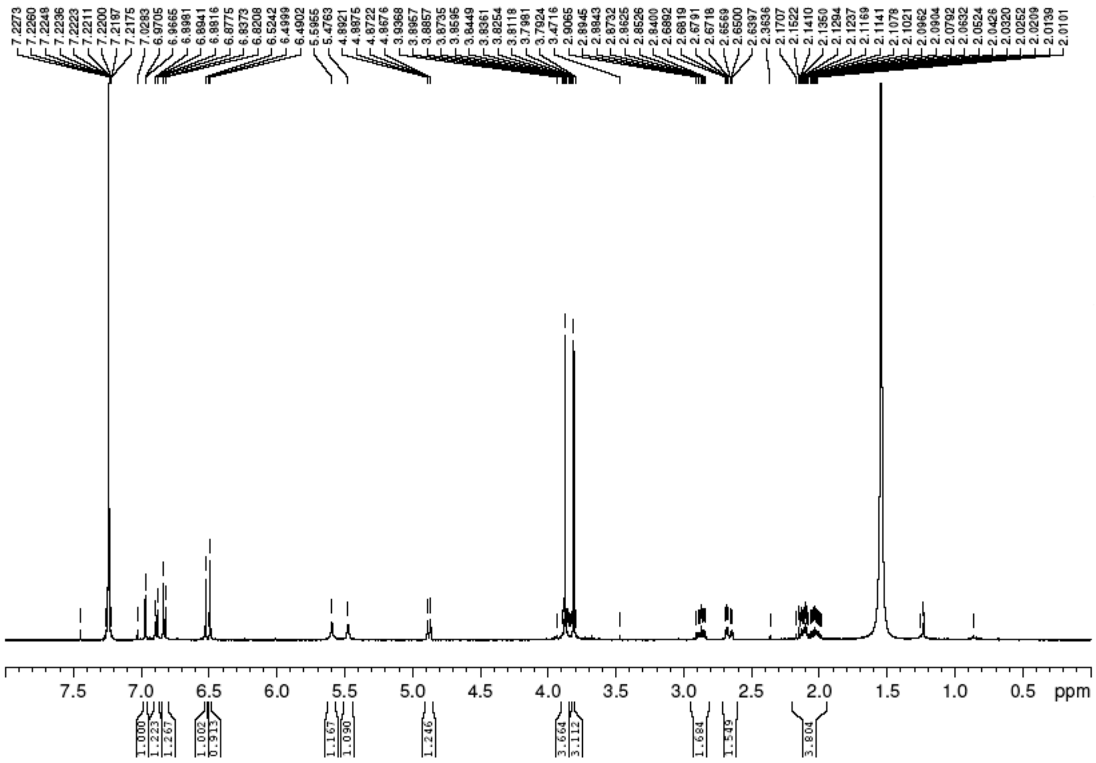

Figure S1. ^{1}H NMR spectrum of **1**

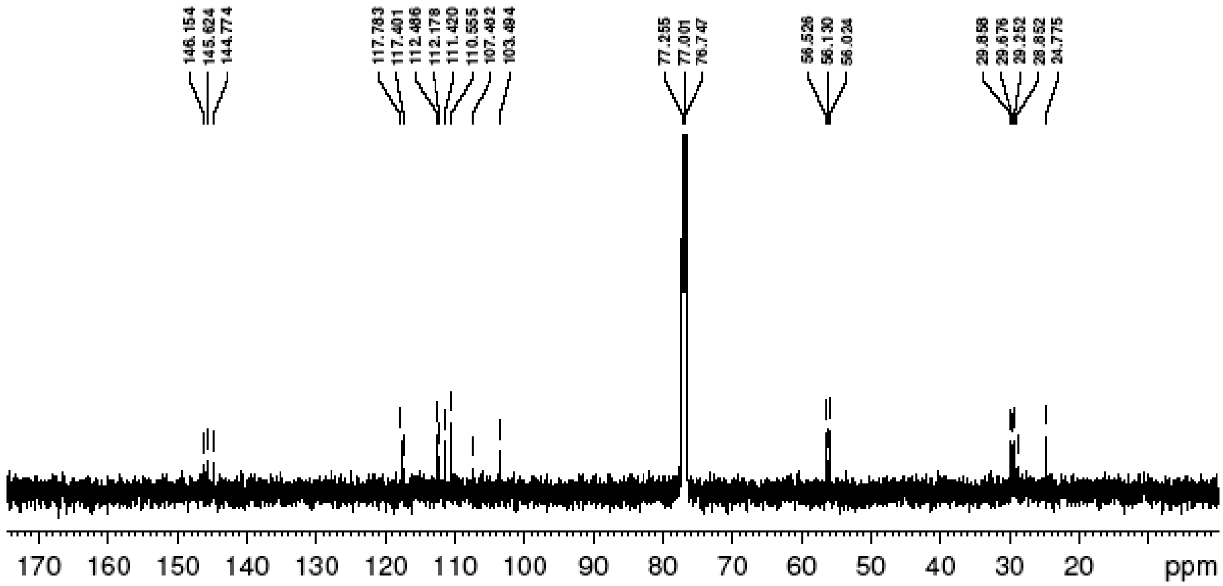

Figure S2. ^{13}C NMR spectrum of **1**

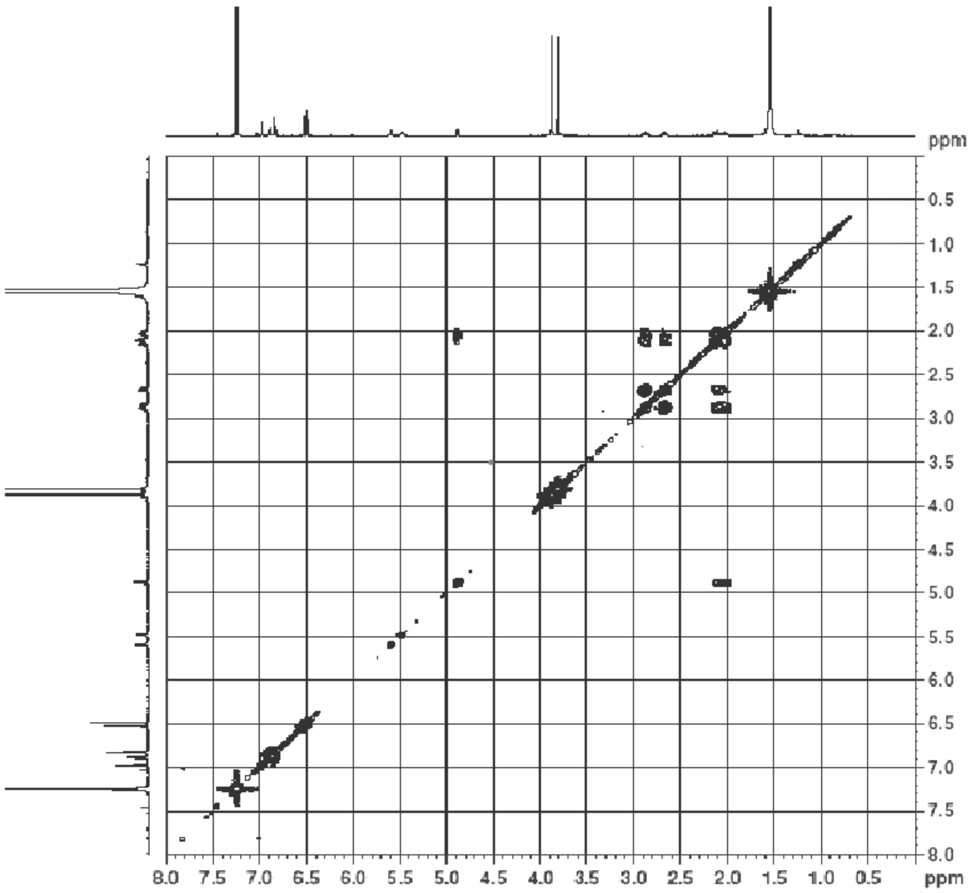

Figure S3. COSY spectrum of **1**

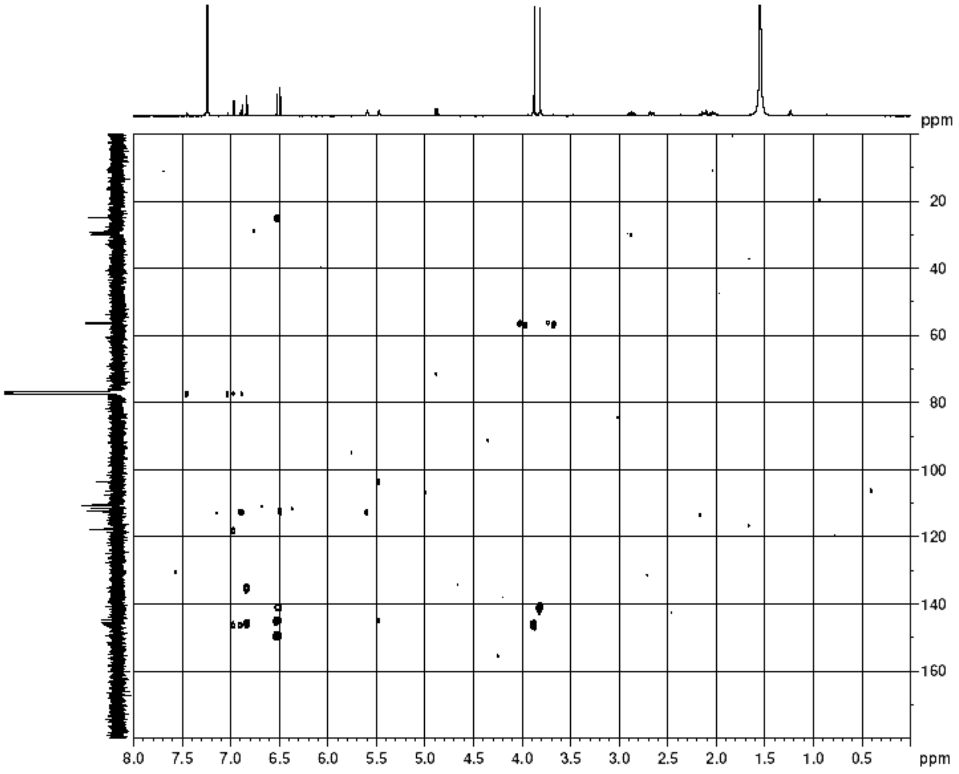

Figure S4. HMBC spectrum of **1**

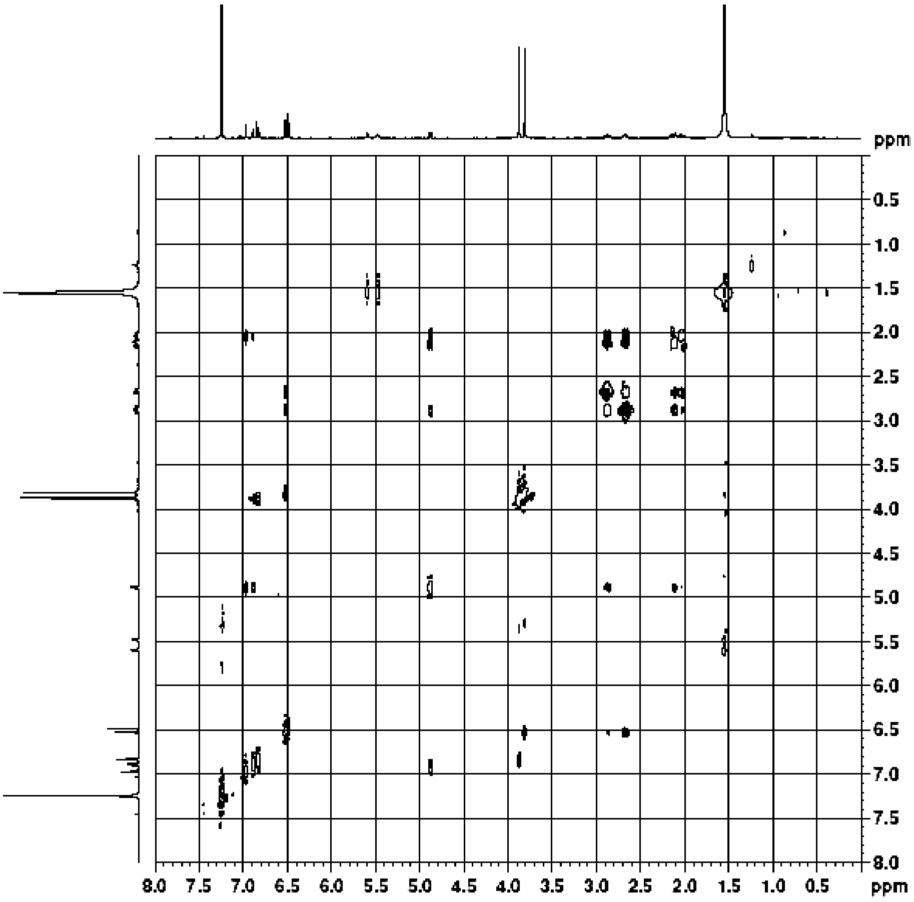

Figure S5. NOESY spectrum of **1**

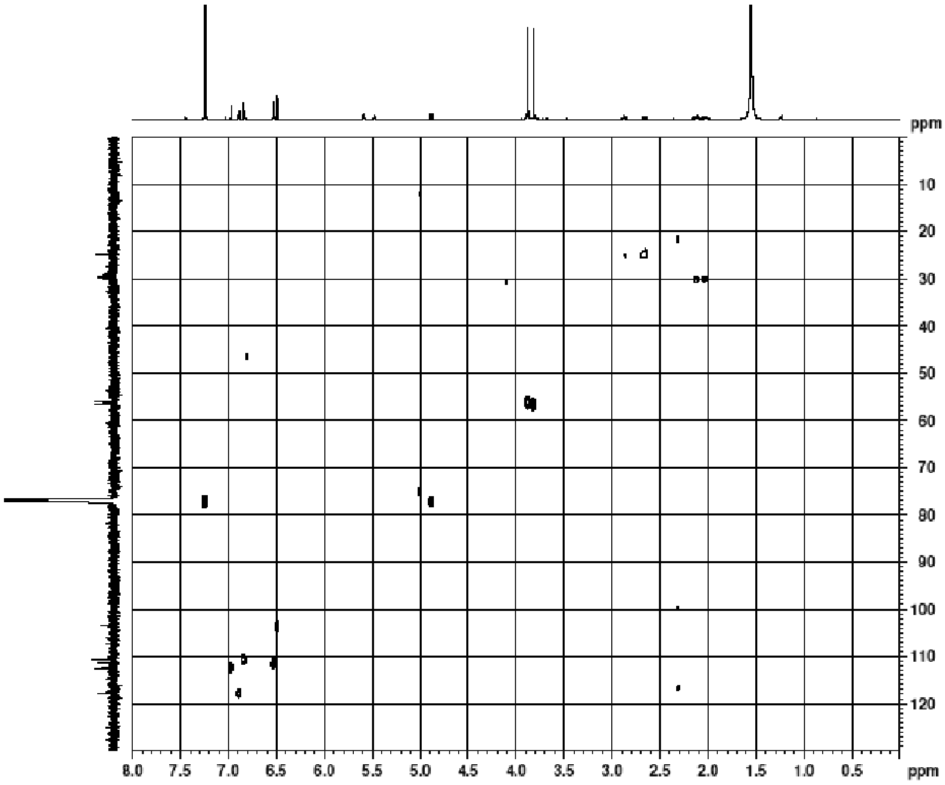

Figure S6. HSQC spectrum of **1**

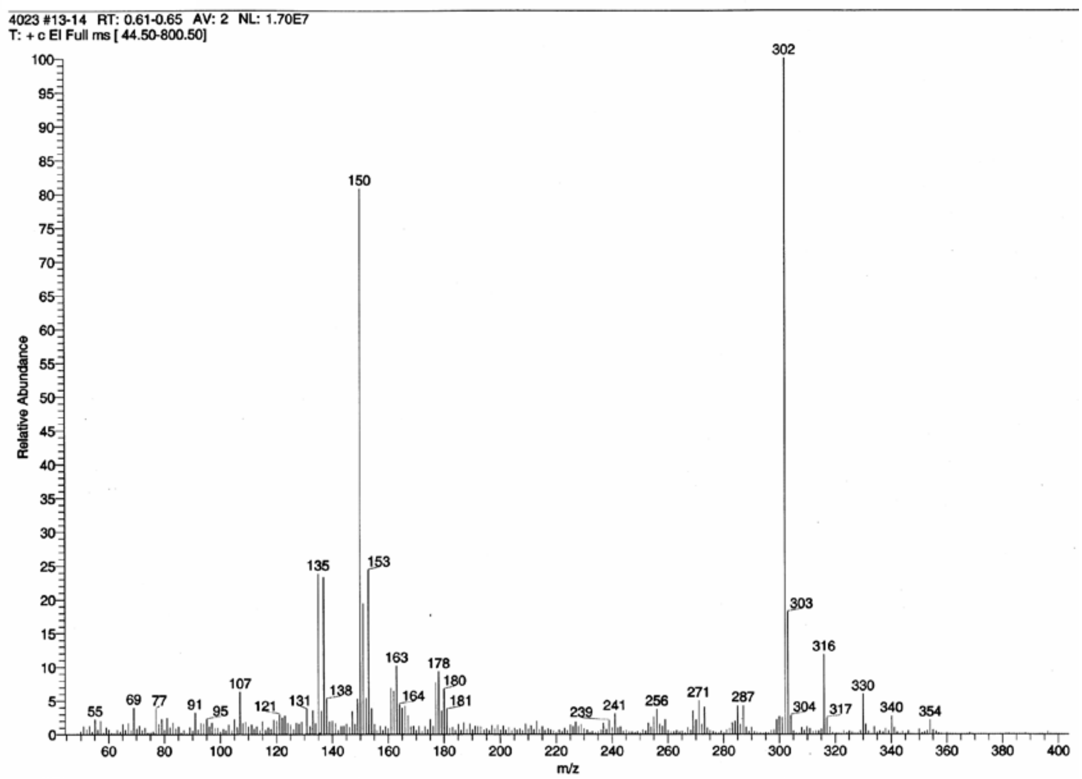

Figure S7. EIMS spectrum of **1**

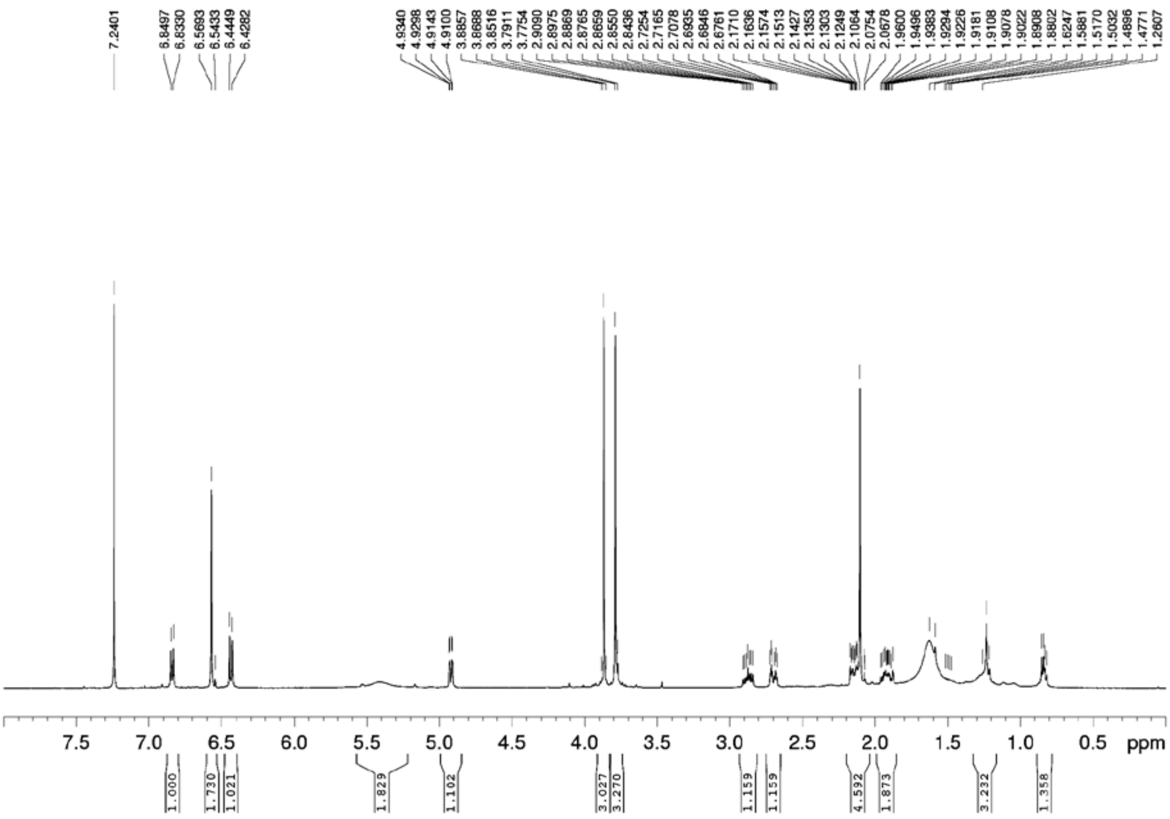

Figure S8. ^{1}H NMR spectrum of **2**

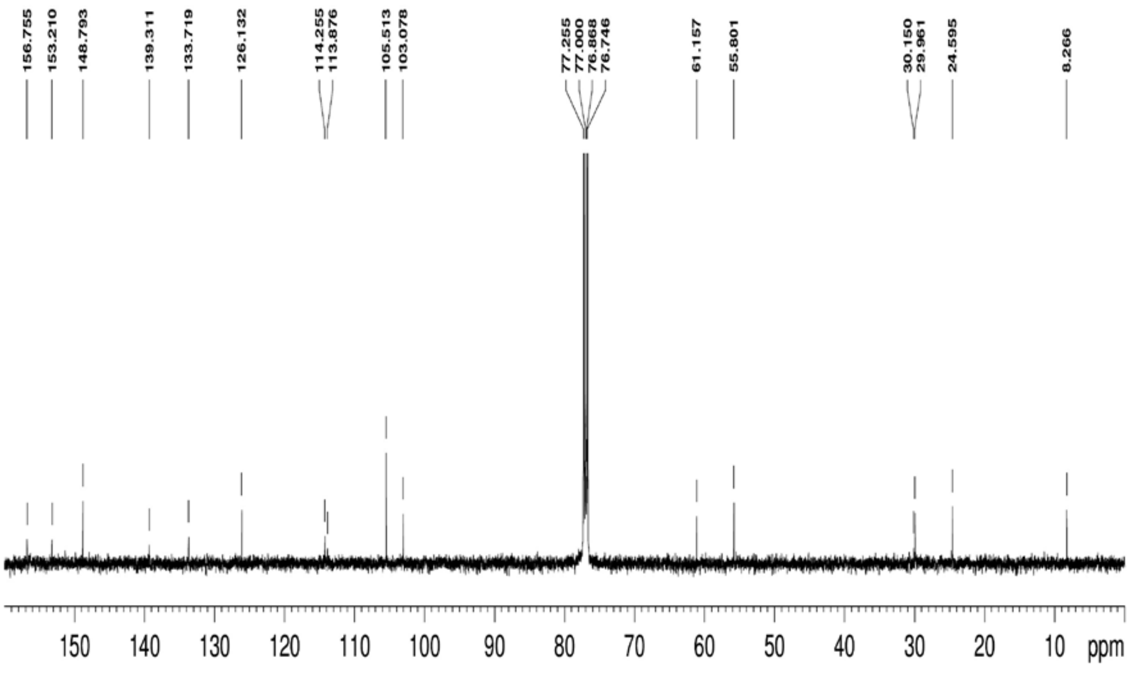

Figure S9. ^{13}C spectrum of **2**

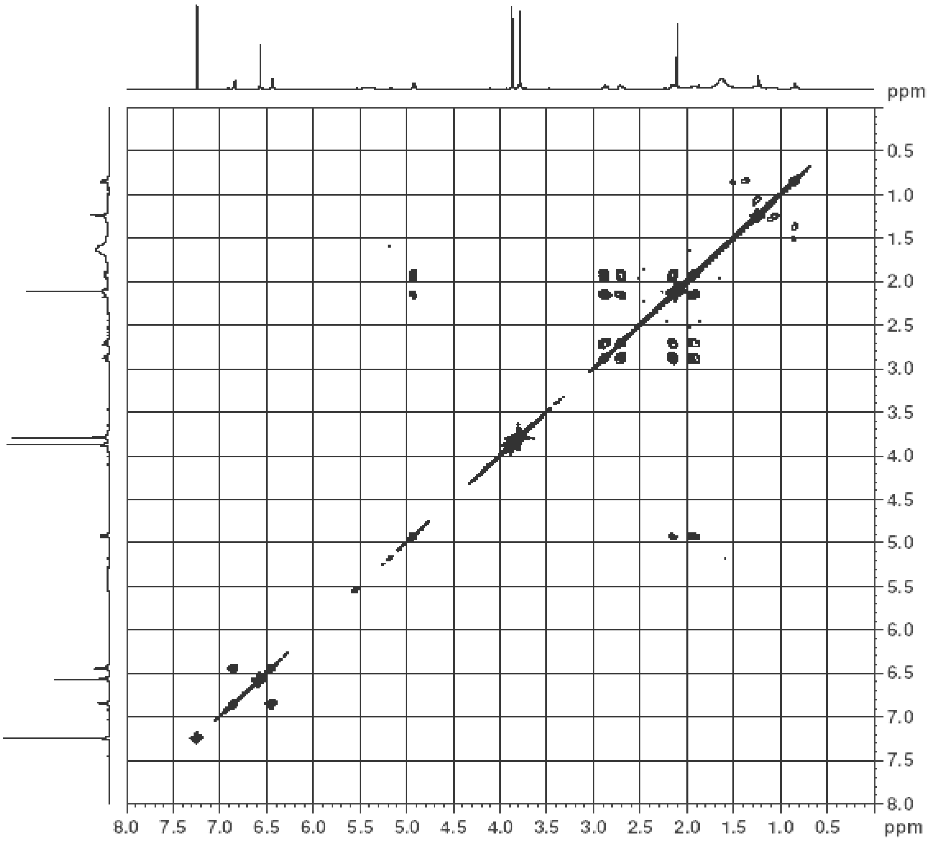

Figure S10. COSY spectrum of **2**

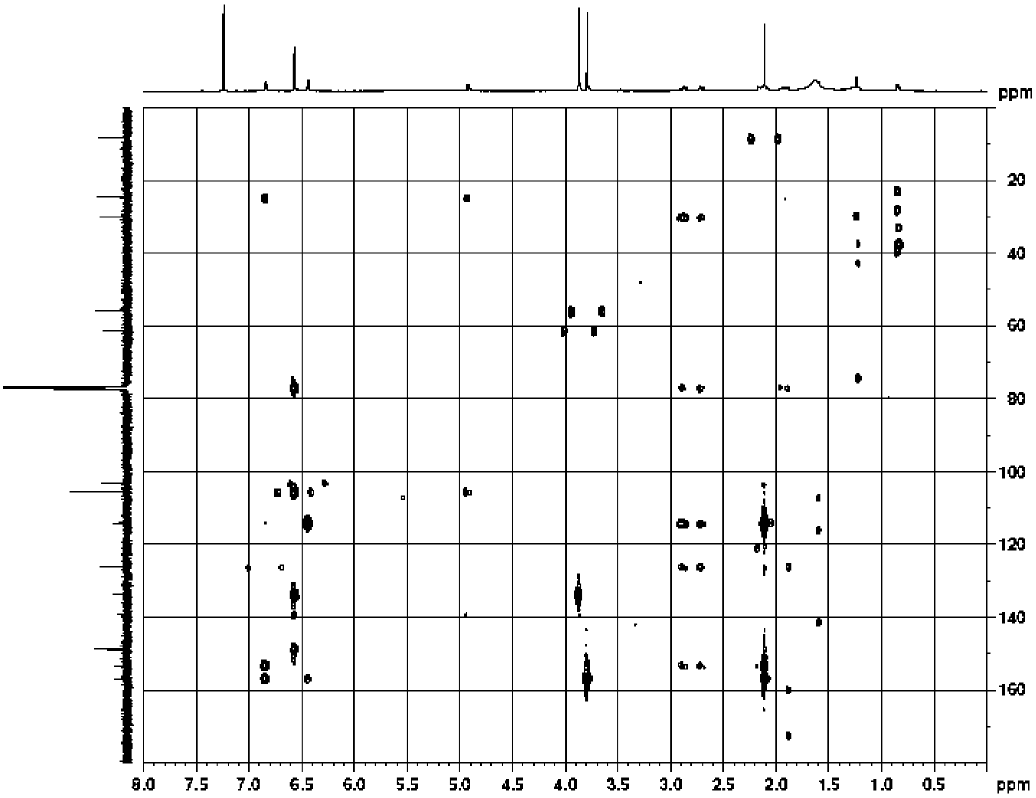

Figure S11. HMBC spectrum of **2**

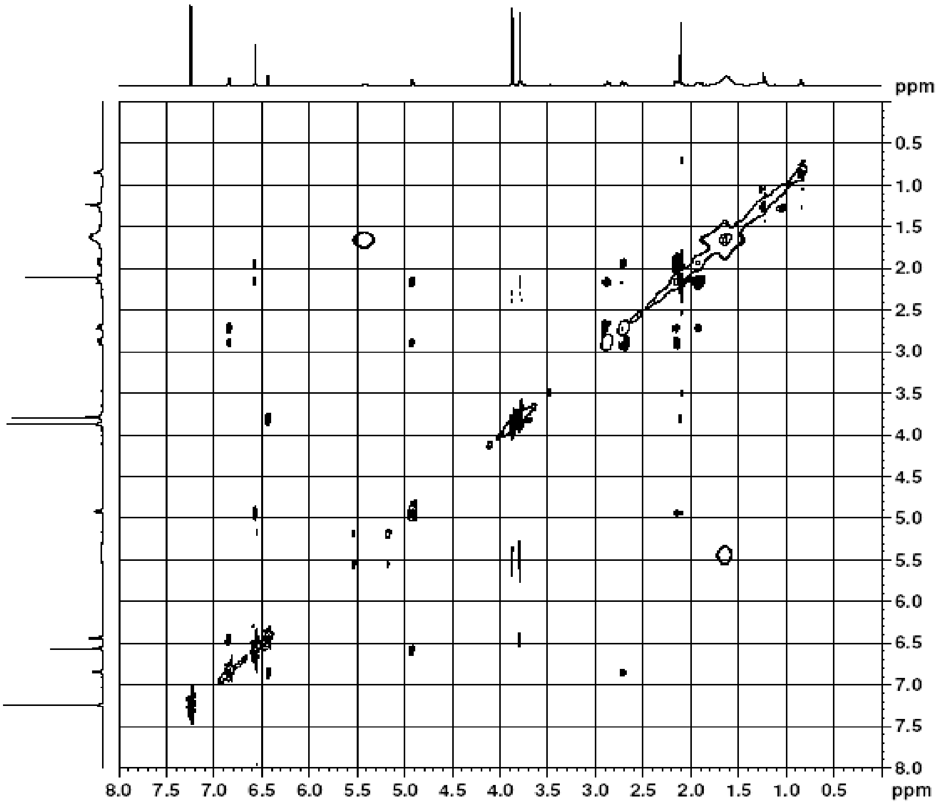

Figure S12. NOESY spectrum of **2**

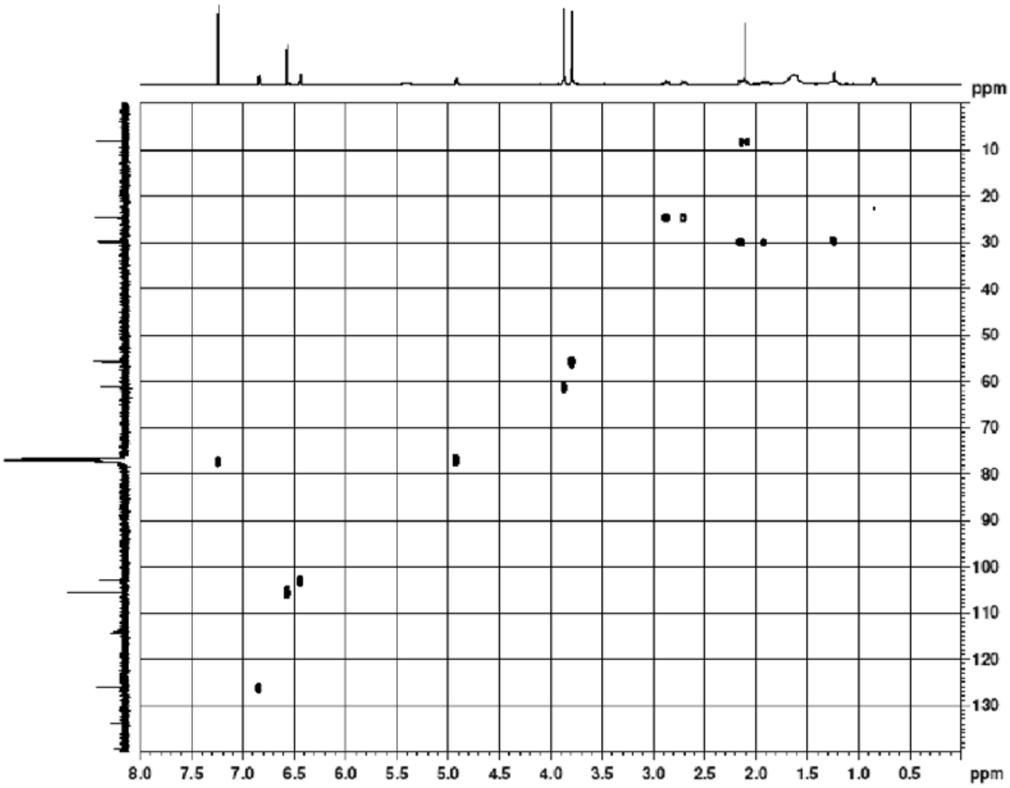

Figure S13. HSQC spectrum of **2**

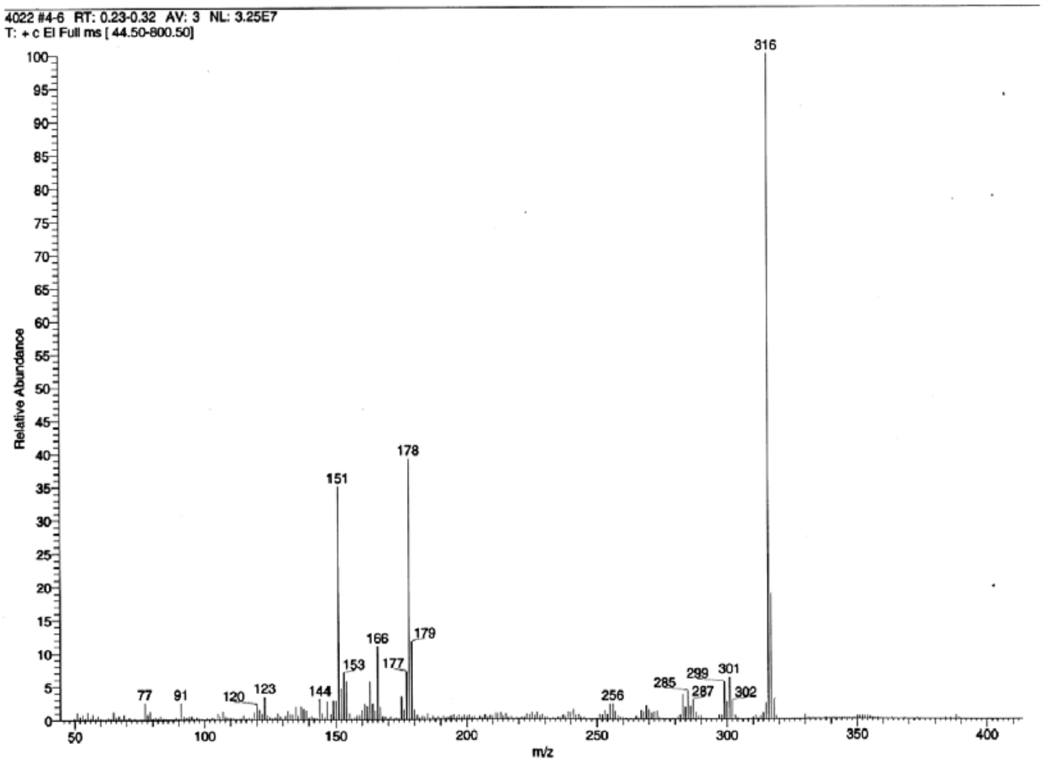

Figure S14. EI-MS spectrum of **2**

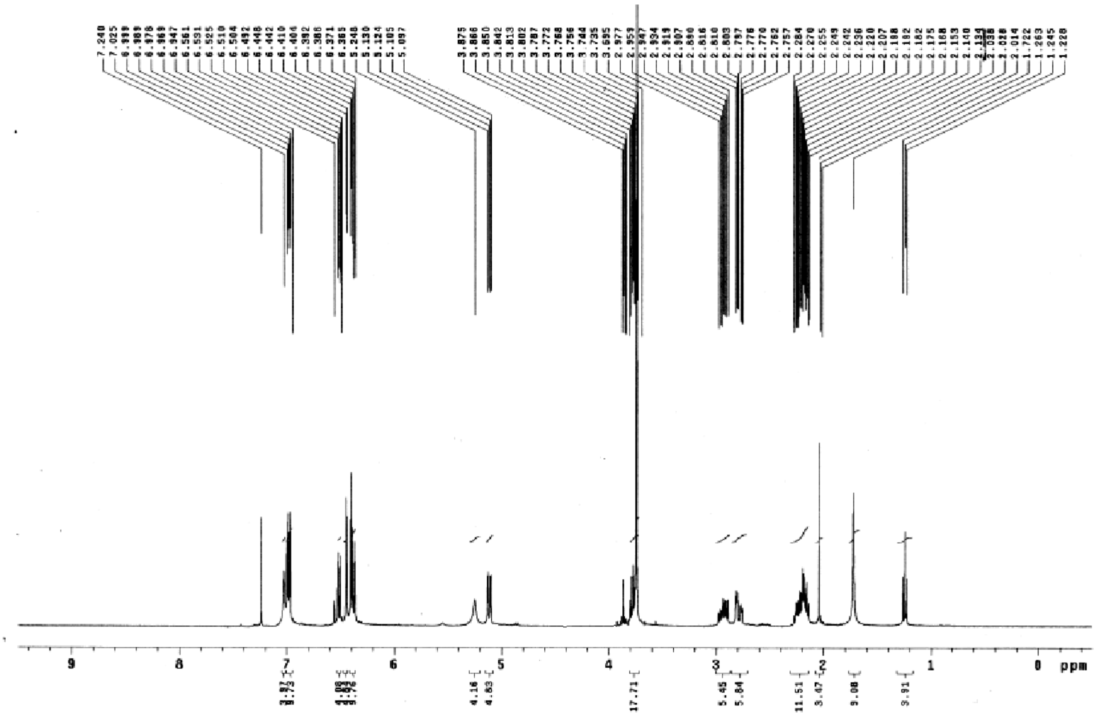

Figure S15. ^{1}H NMR spectrum of **3**

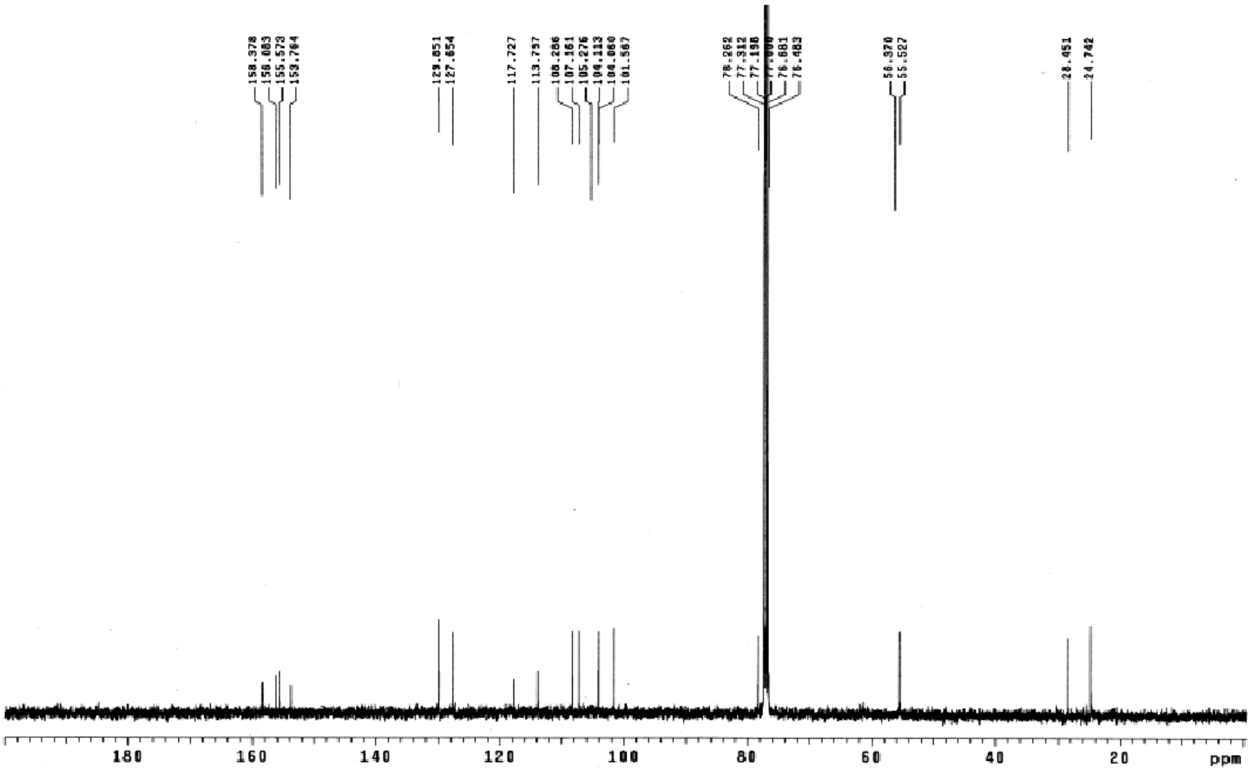

Figure S16. ^{13}C NMR spectrum of **3**

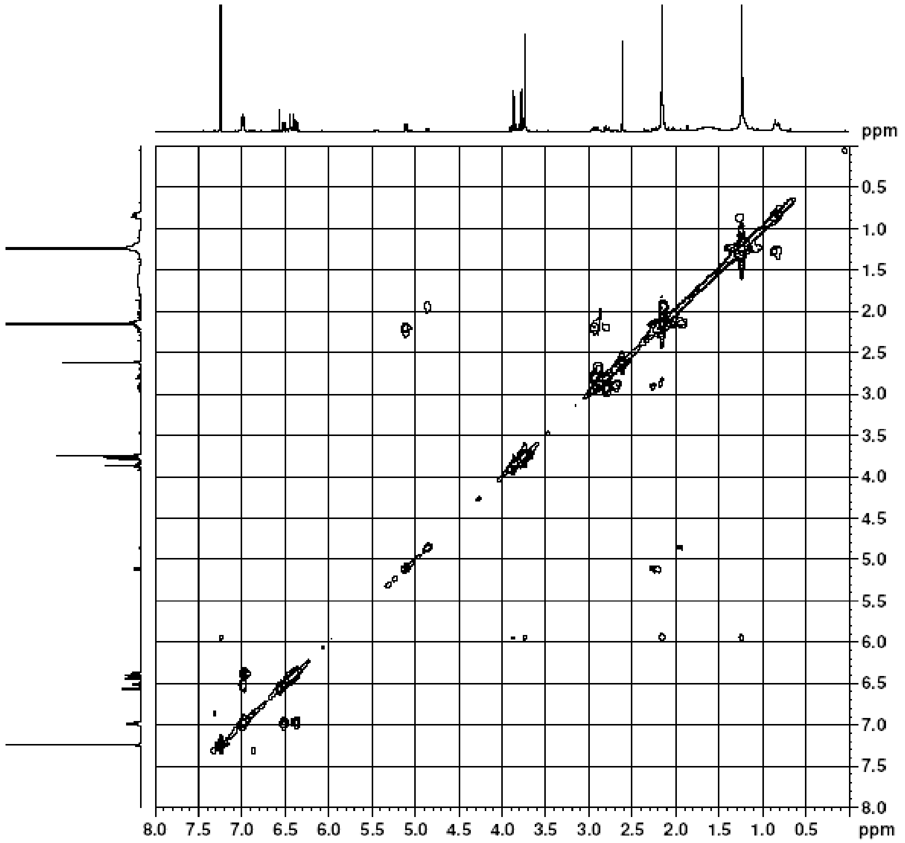

Figure S17. COSY spectrum of **3**

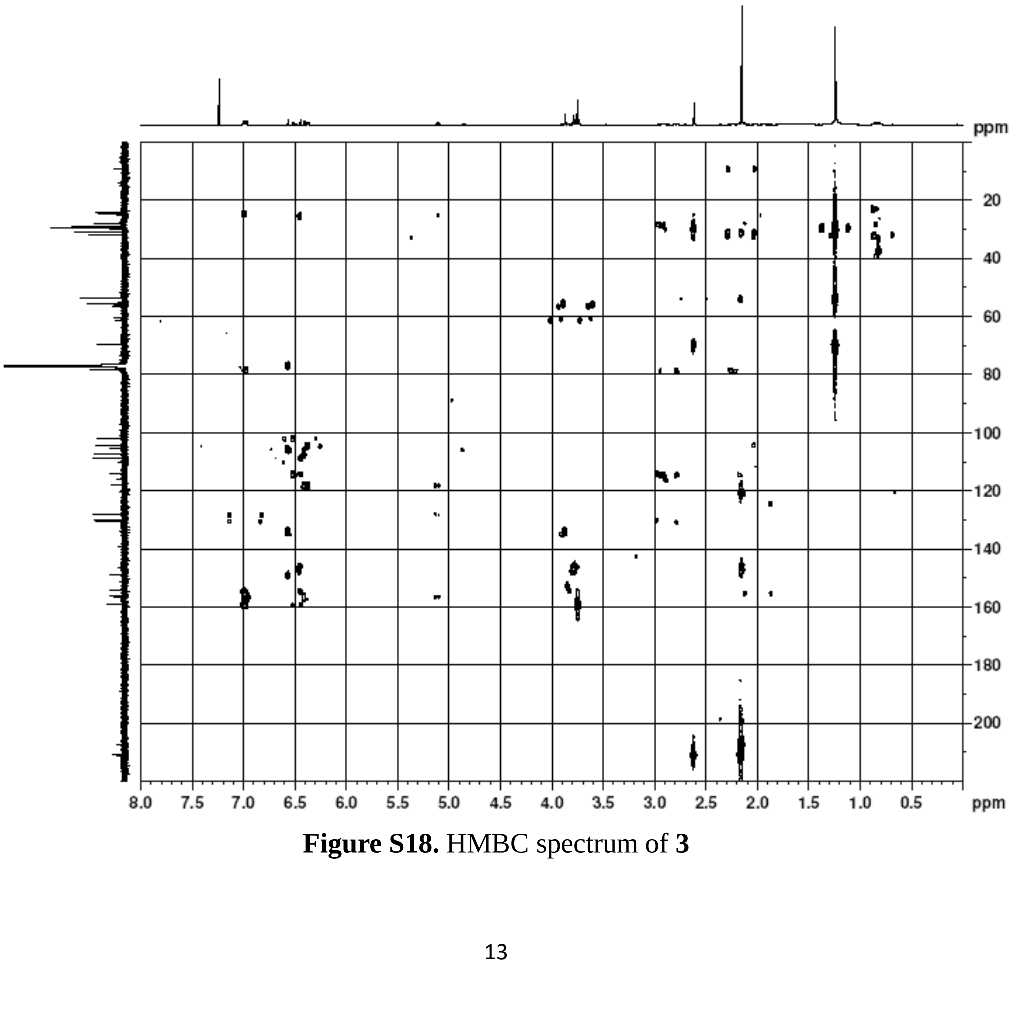

Figure S18. HMBC spectrum of **3**

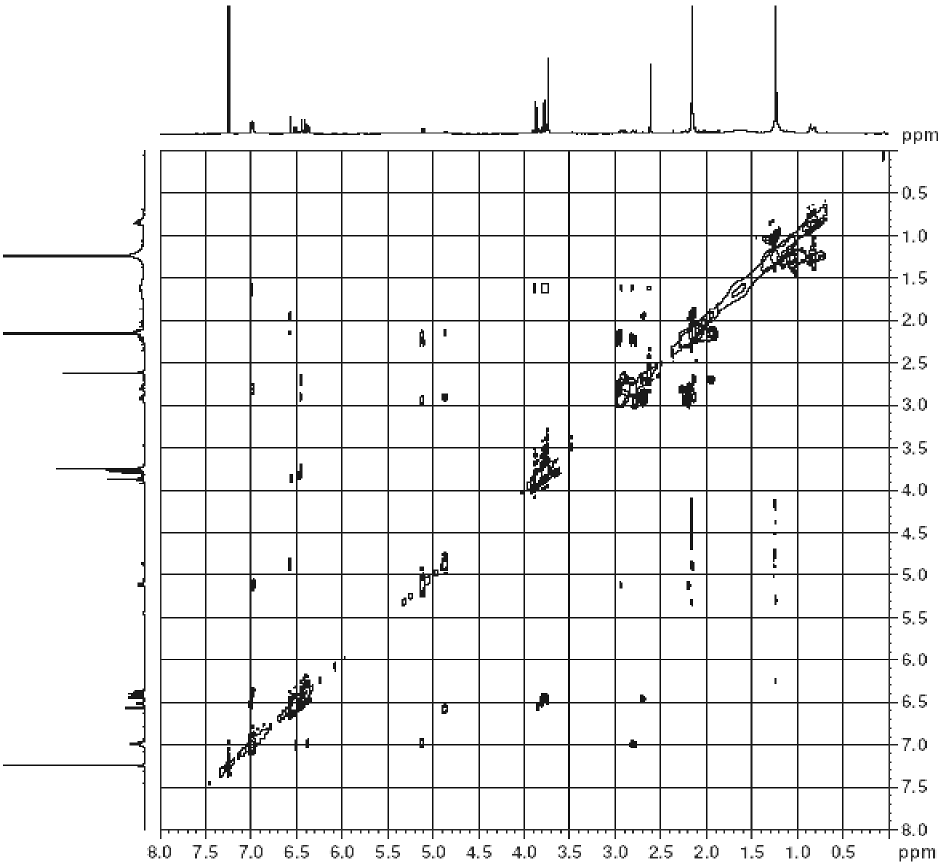

Figure S19. NOESY spectrum of **3**

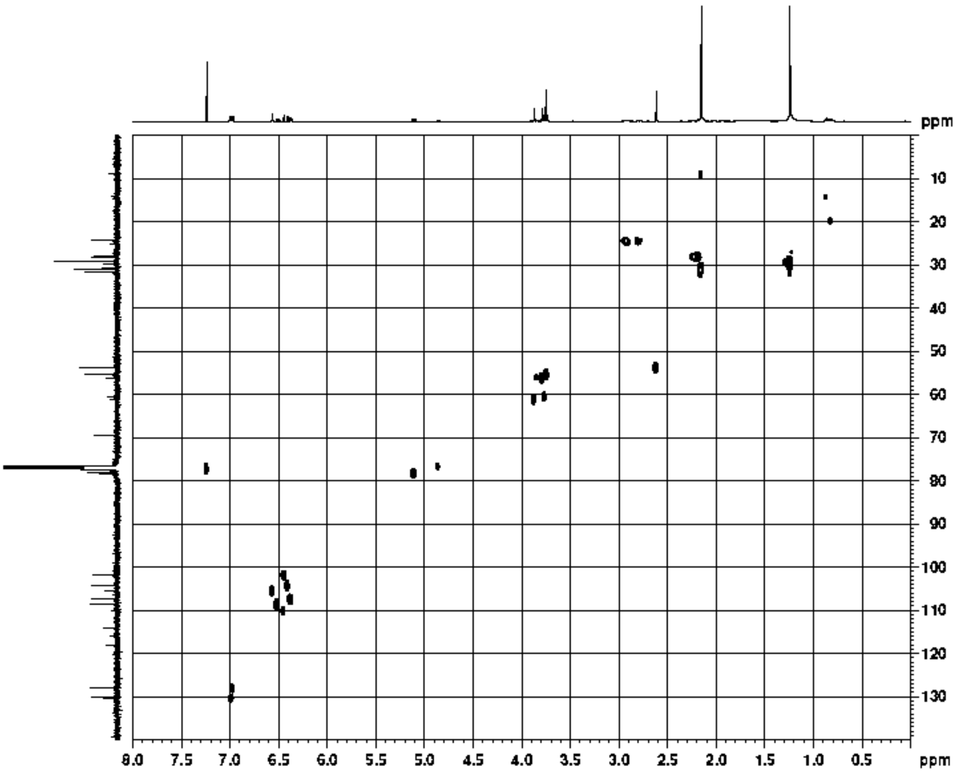

Figure S20. HSQC spectrum of **3**

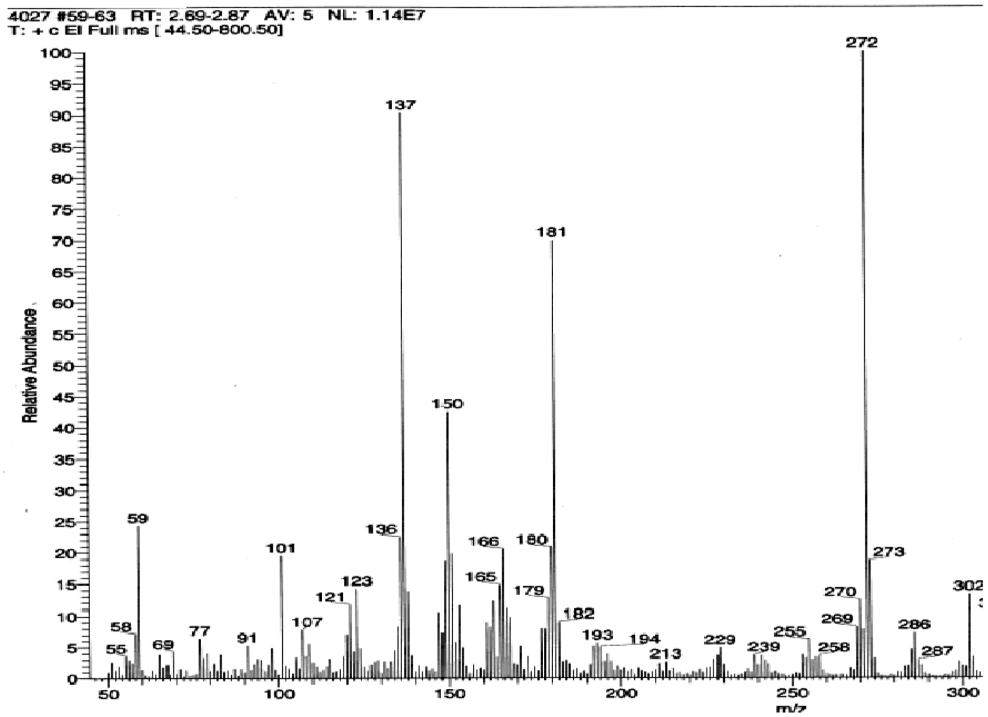

Figure S21. EI-MS spectrum of **3**

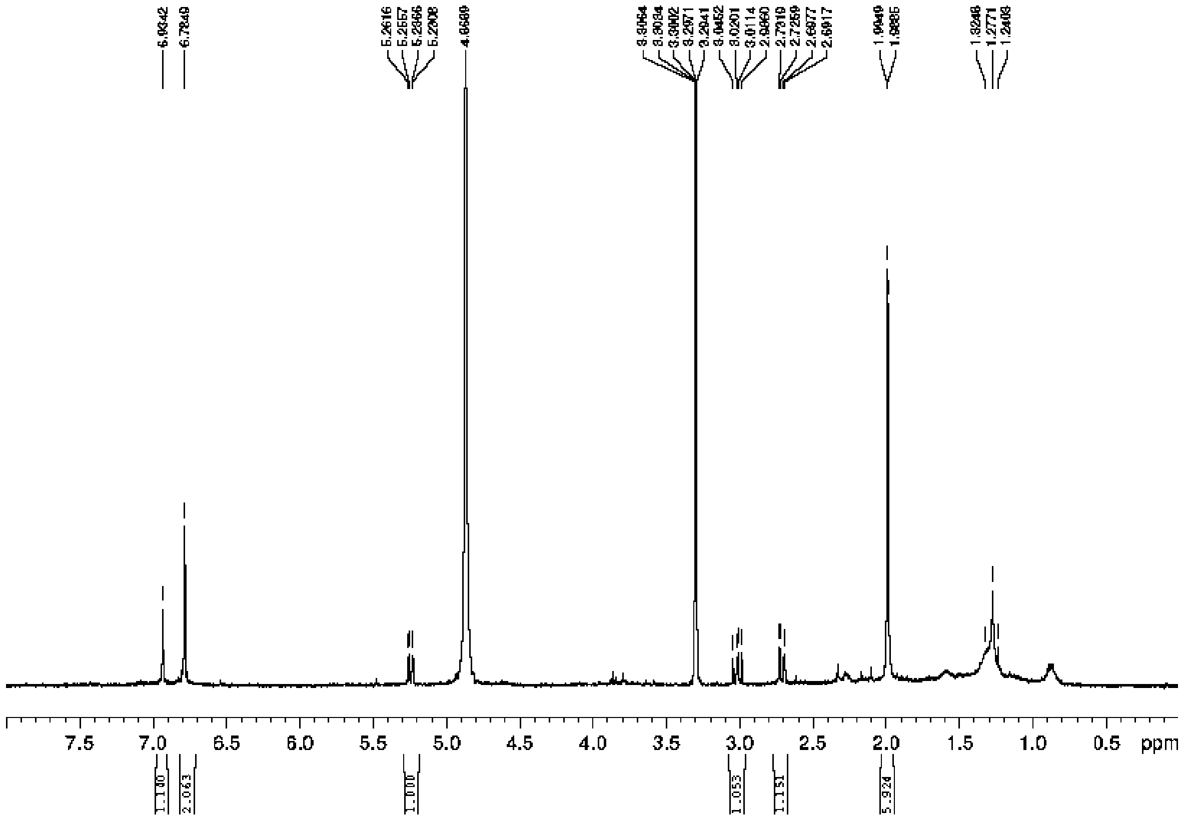

Figure S22. ^{1}H NMR spectrum of **4**

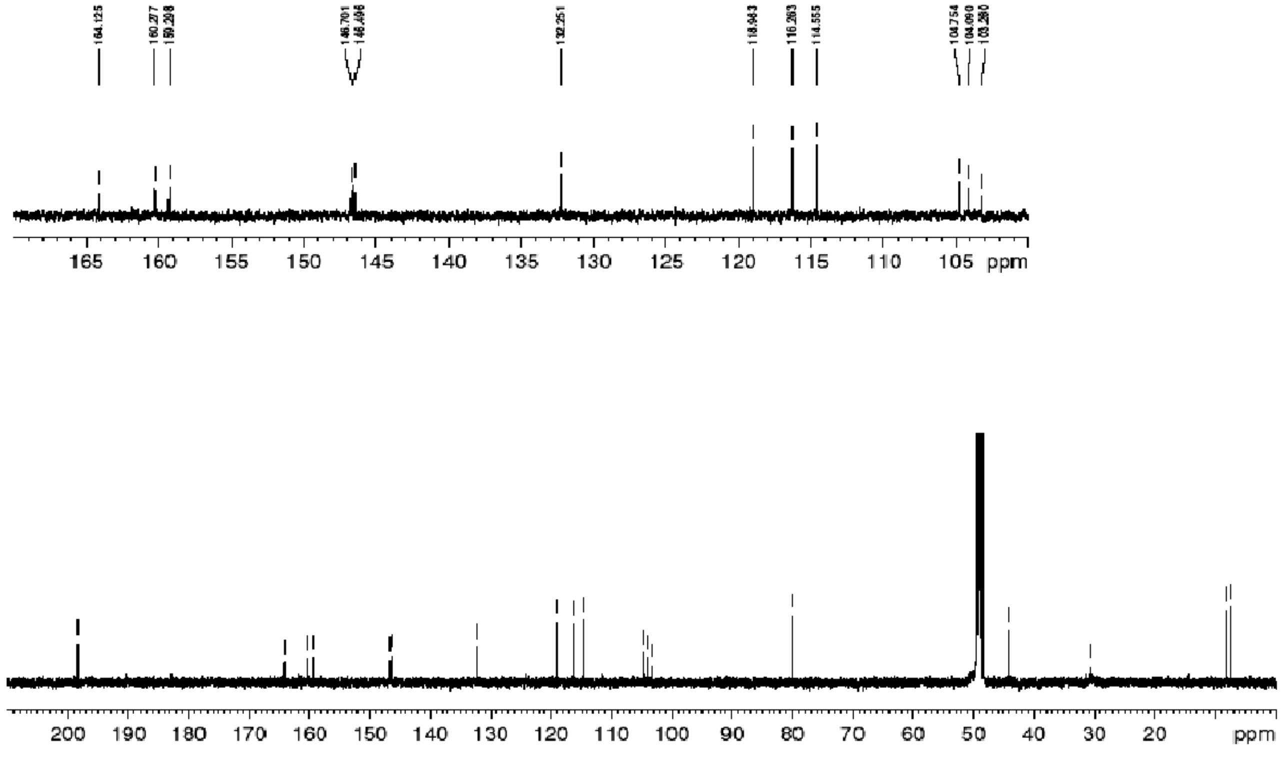

Figure S23. ^{13}C spectrum of **4**

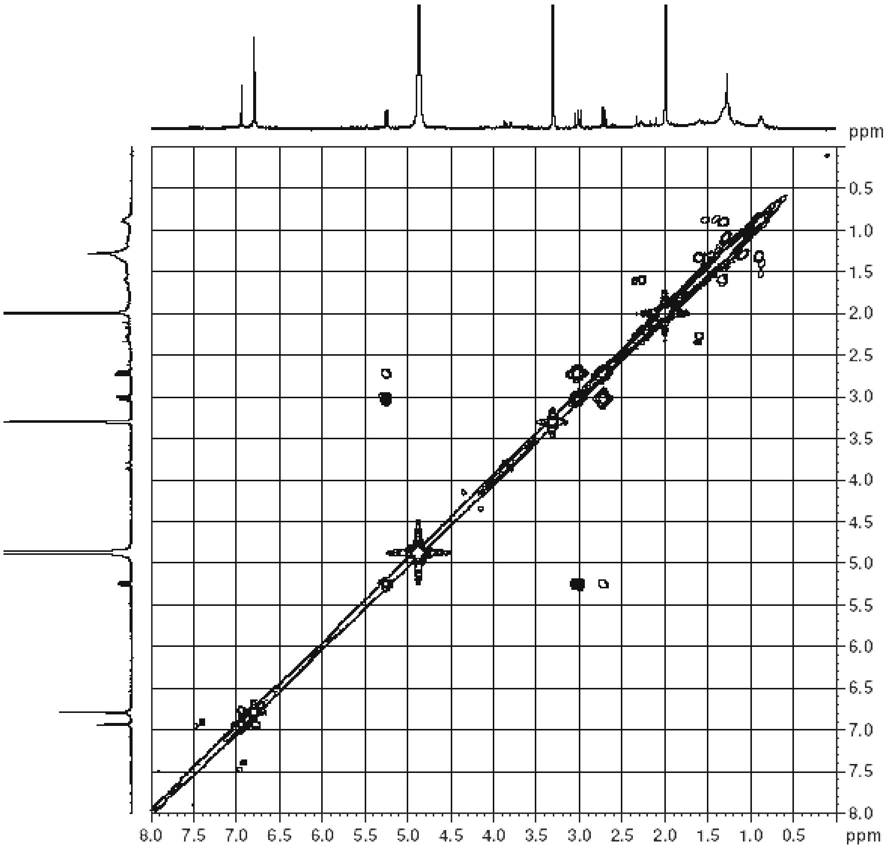

Figure S24. COSY spectrum of **4**

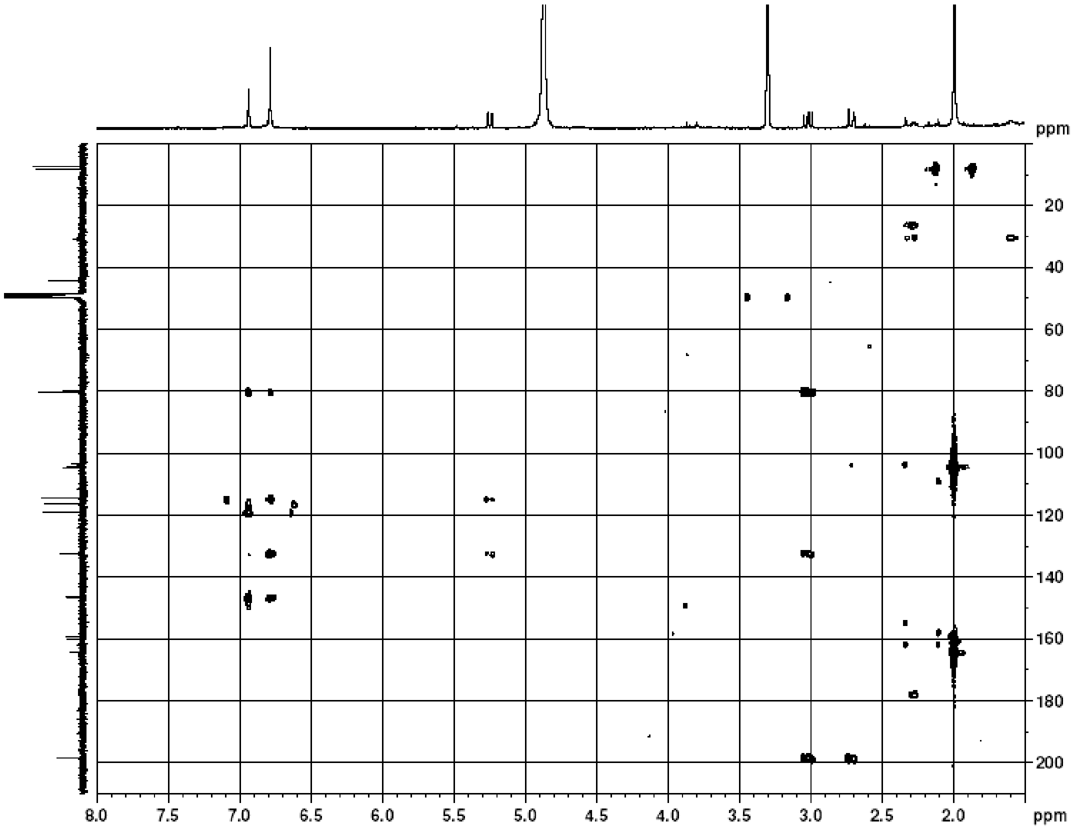

Figure S25. HMBC spectrum of **4**

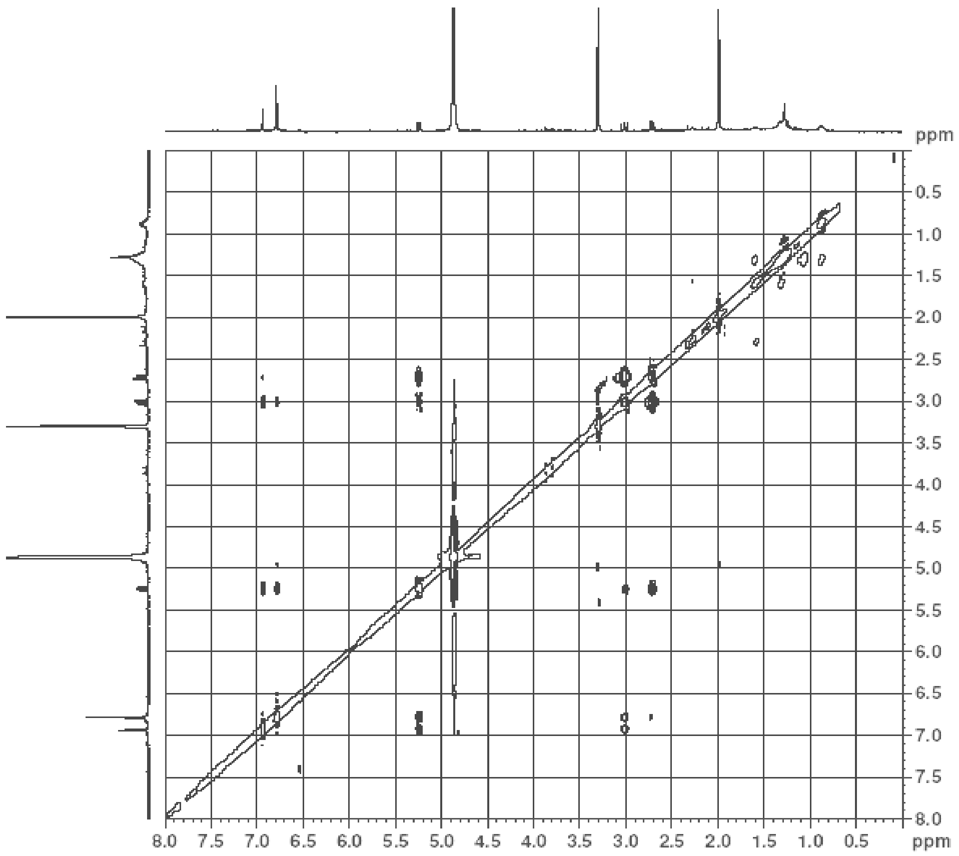

Figure S26. NOESY spectrum of **4**

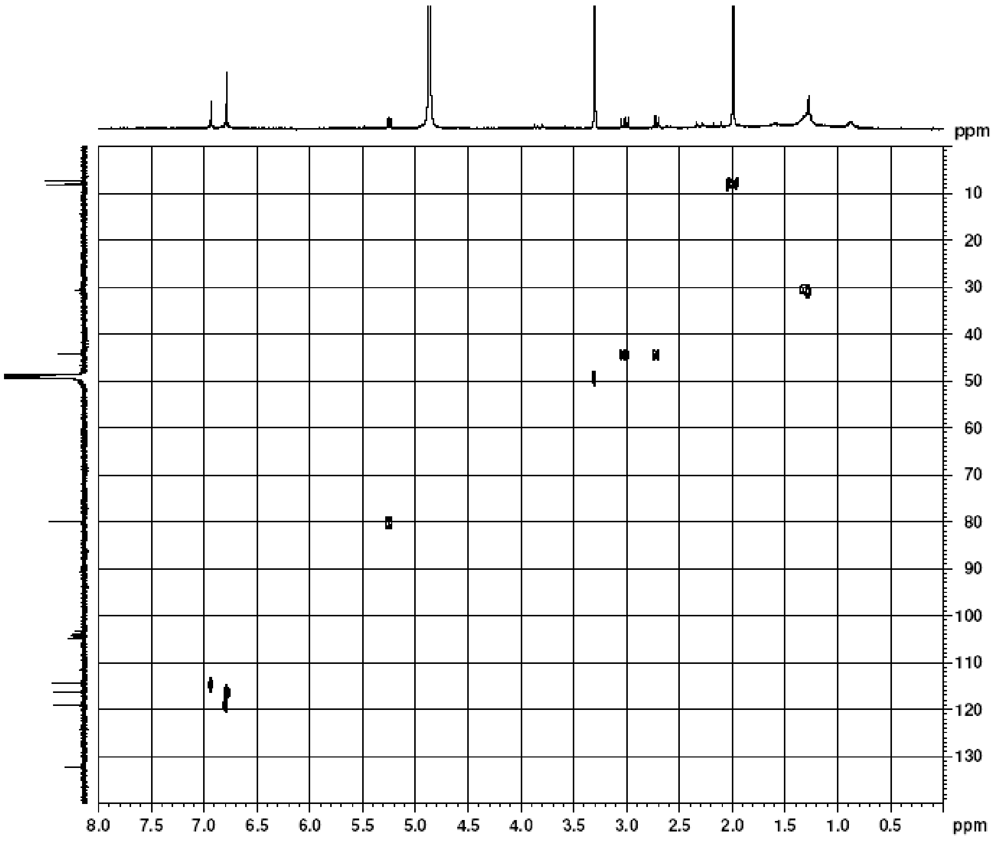

Figure S27. HSQC spectrum of **4**

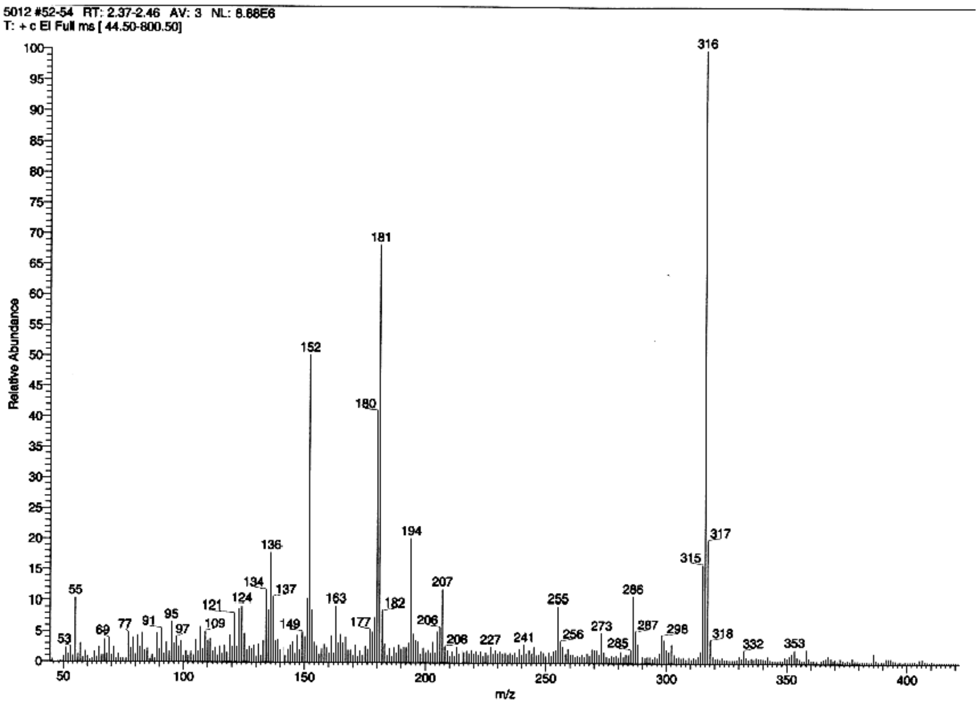

Figure S28. EI-MS spectrum of **4**

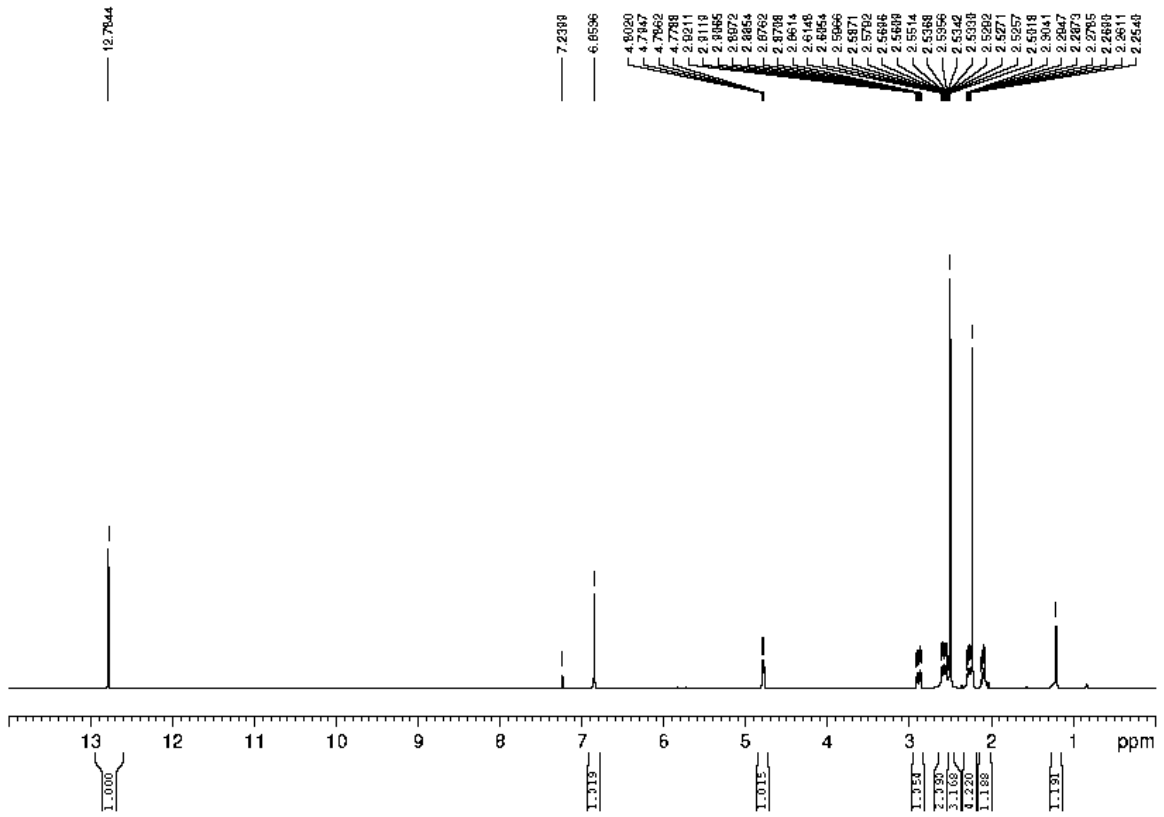

Figure S29. ^{1}H NMR spectrum of **5**

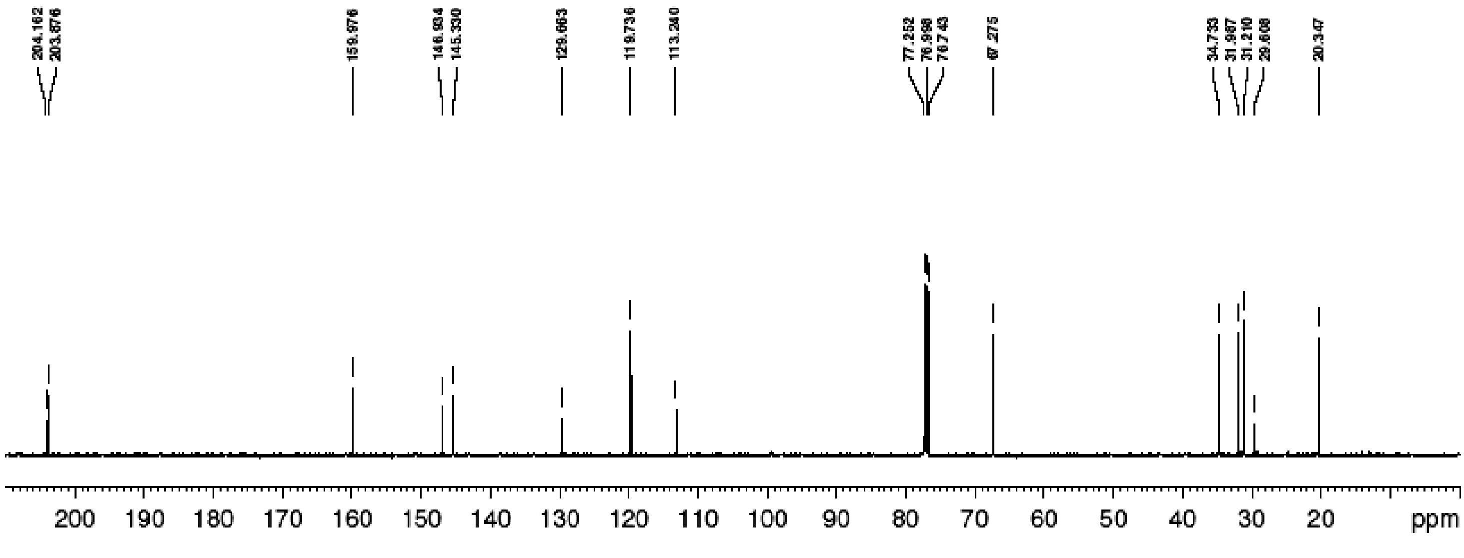

Figure S30. ^{13}C spectrum of **5**

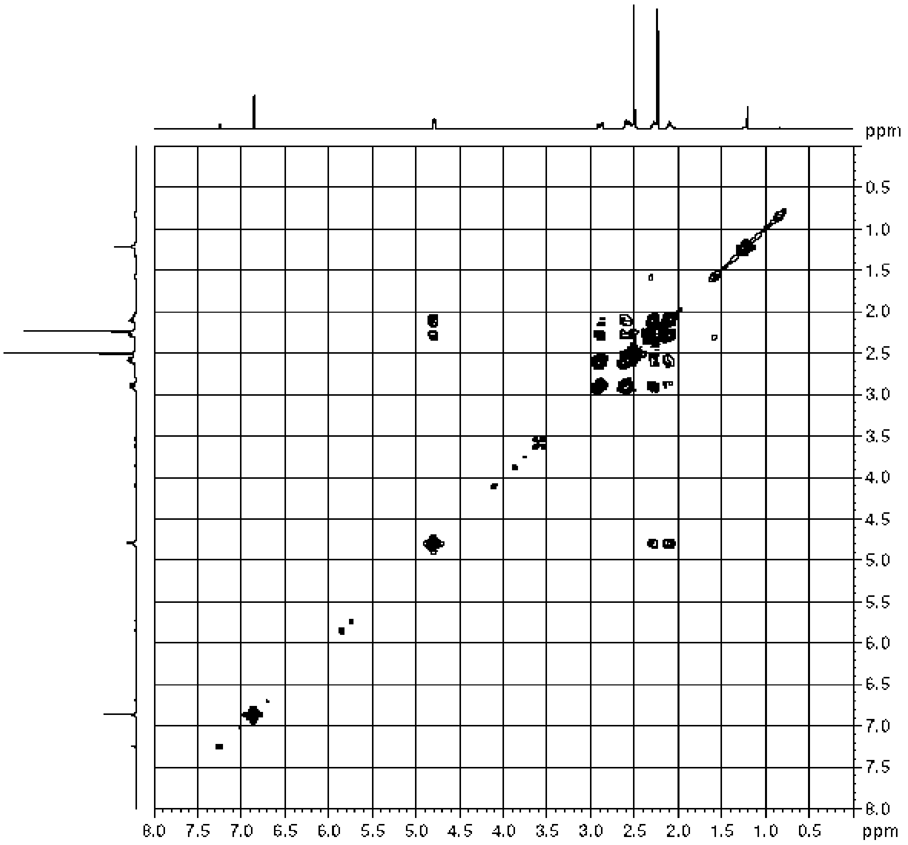

Figure S31. COSY spectrum of **5**

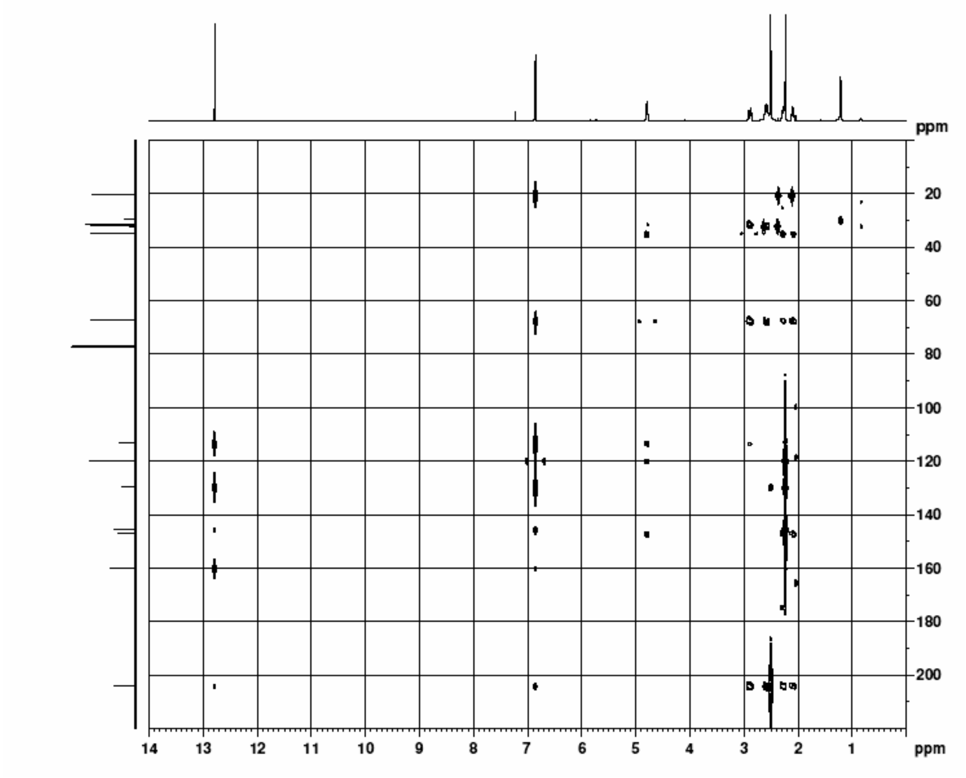

Figure S32. HMBC spectrum of **5**

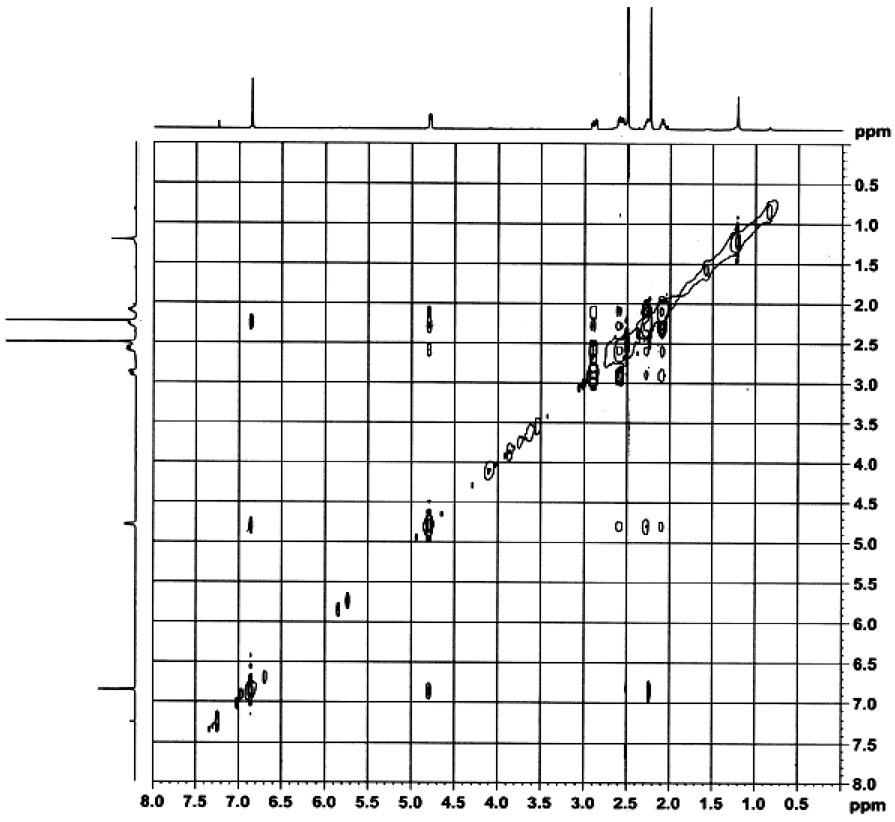

Figure S33. NOESY spectrum of **5**

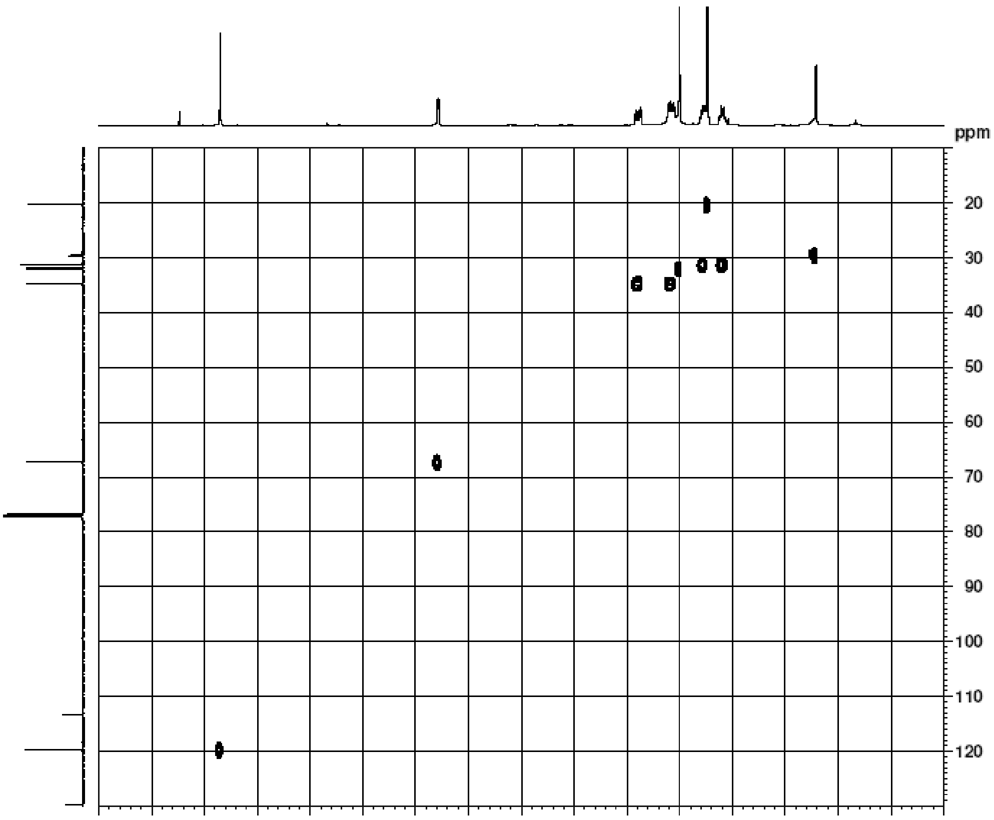

Figure S34. HSQC spectrum of **5**

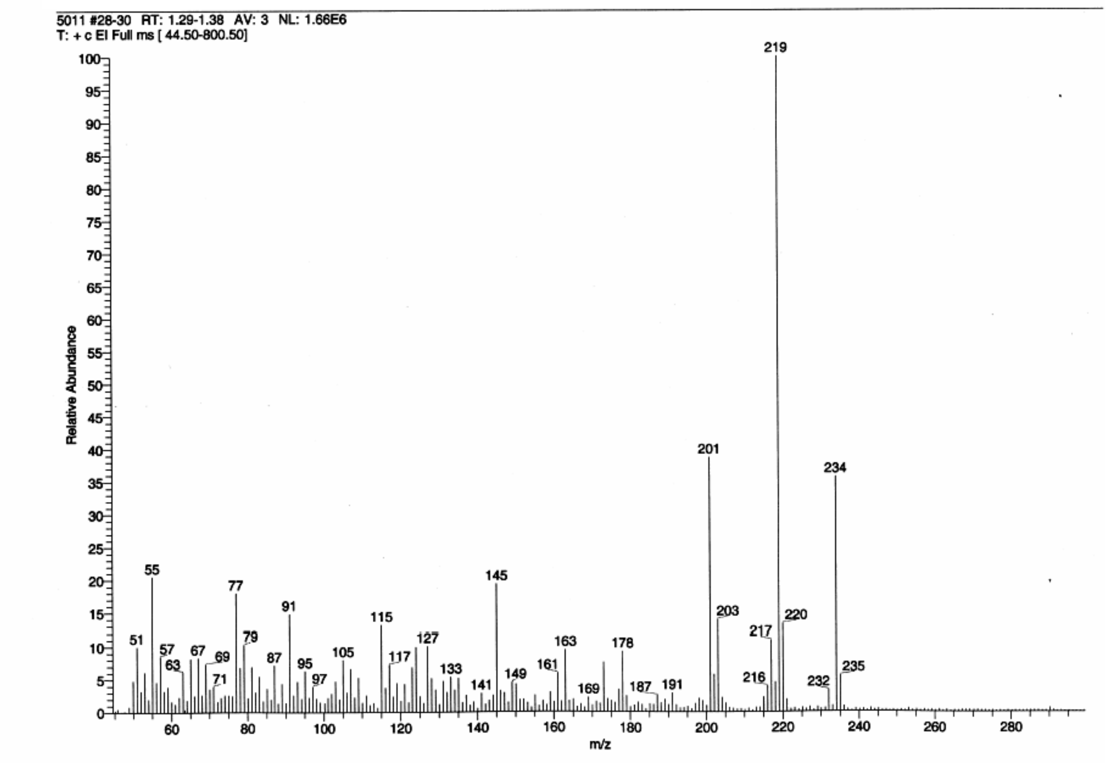

Figure S35. EI-MS spectrum of **5**

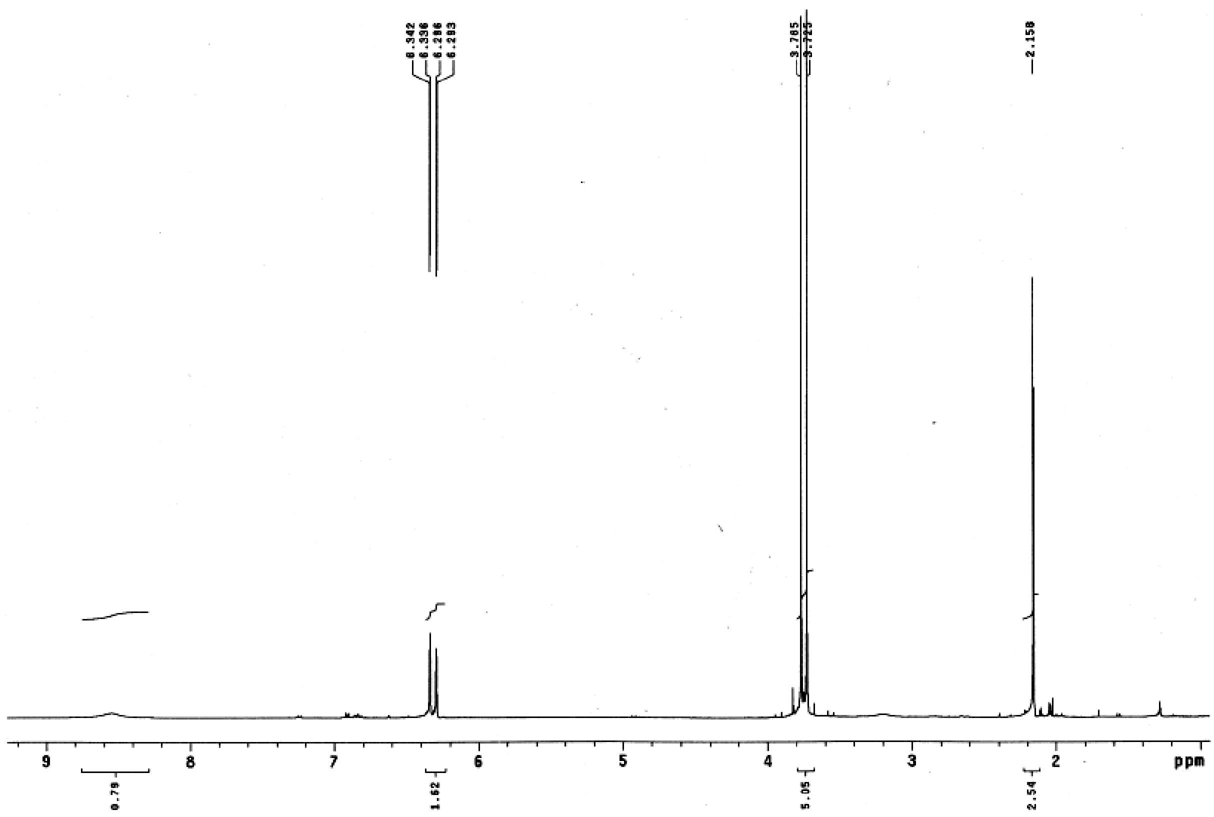

Figure S36. ^{1}H NMR spectrum of **6**

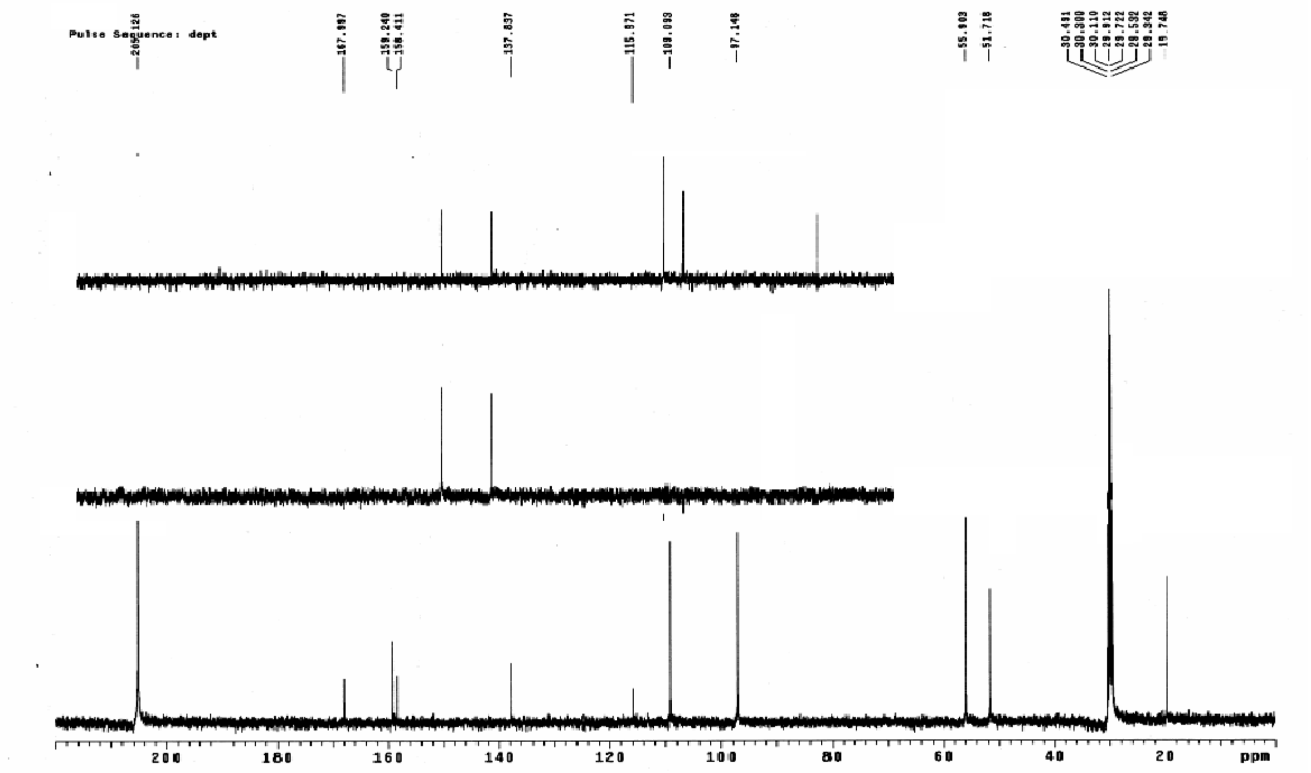

Figure S37. ^{13}C NMR/DEPT spectra of **6**

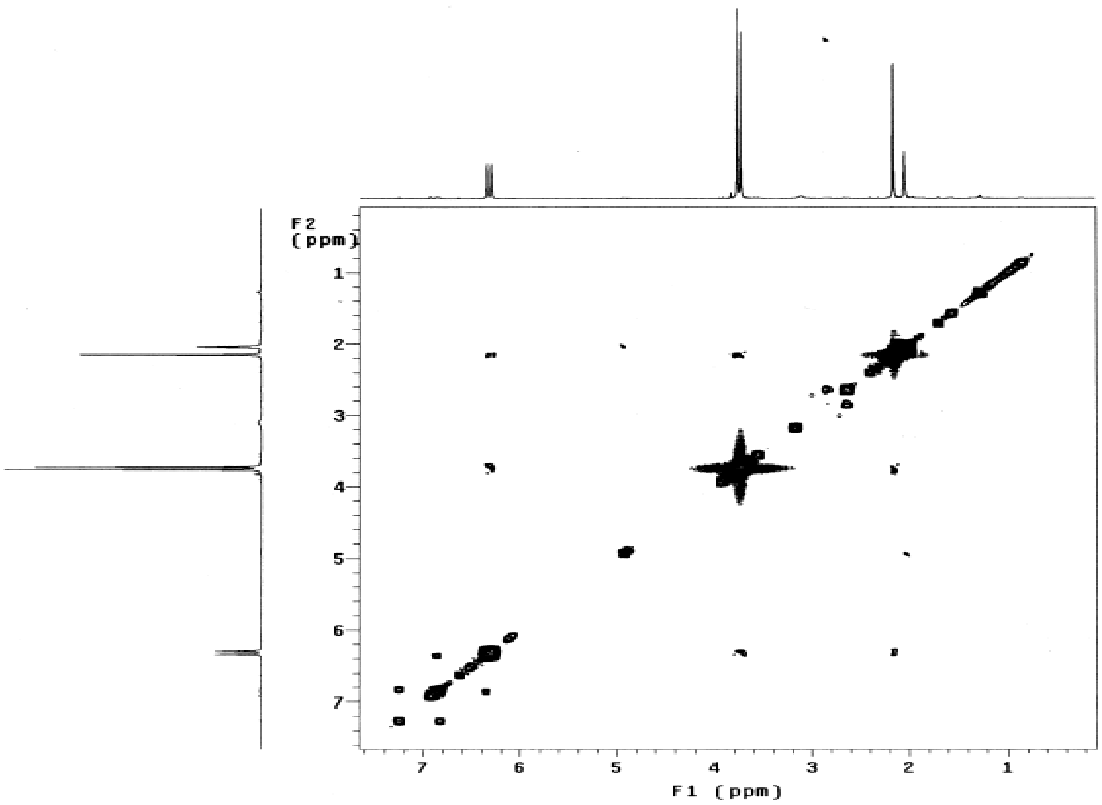

Figure S38. COSY spectrum of **6**

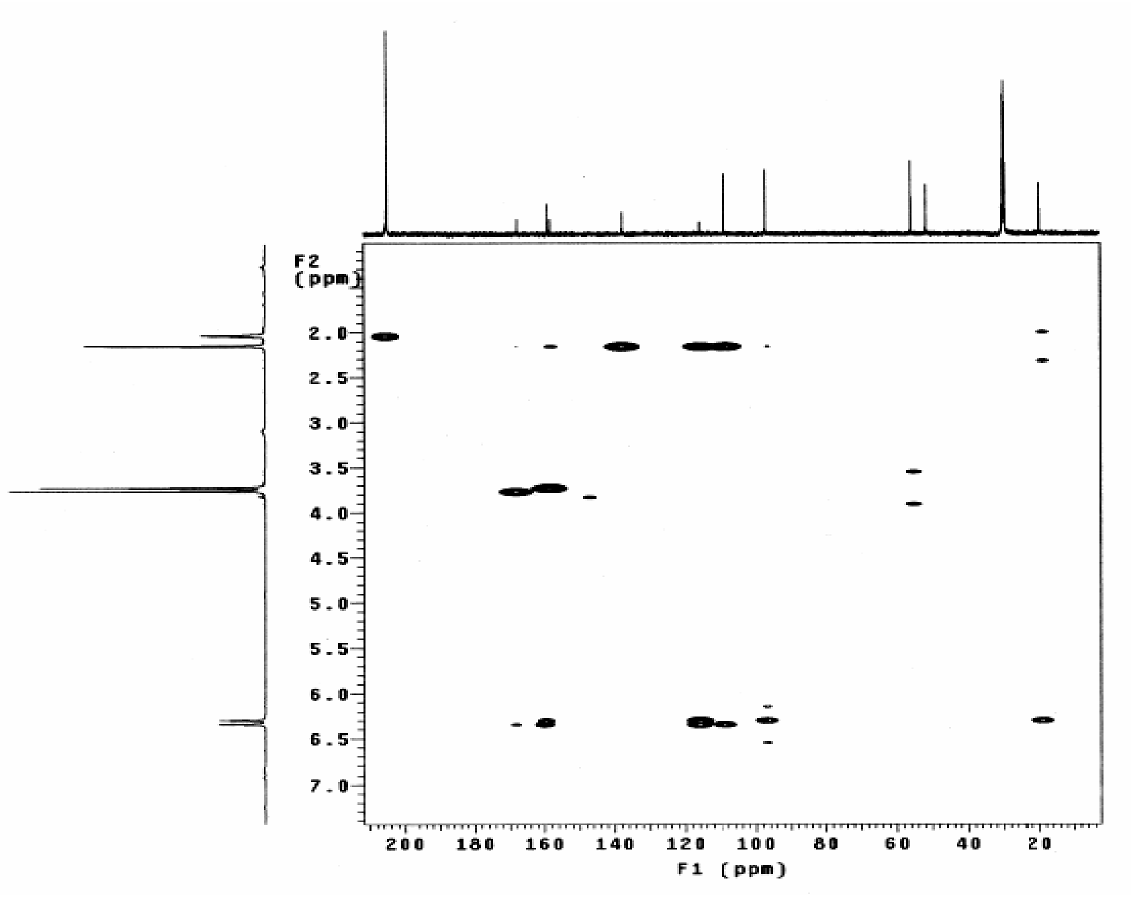

Figure S39. HMBC spectrum of **6**

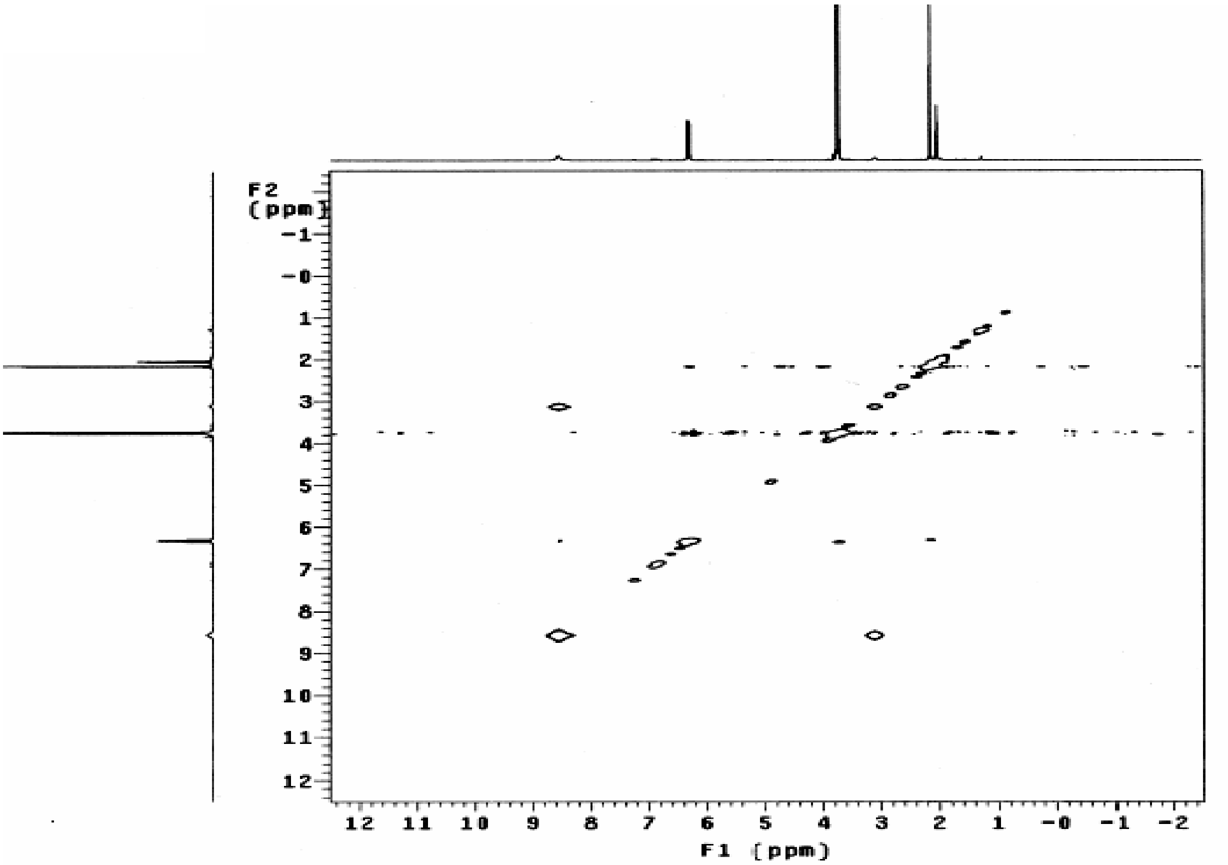

Figure S40. NOESY spectrum of **6**

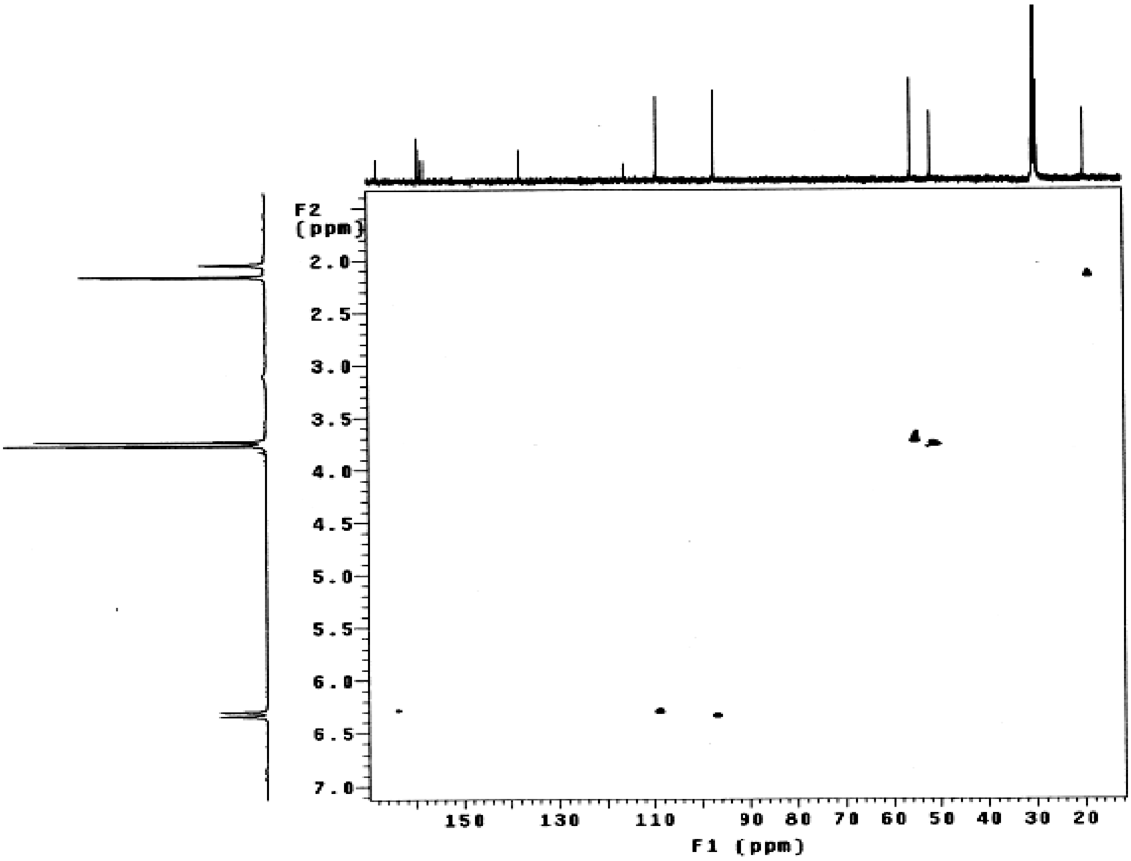

Figure S41. HSQC spectrum of **6**

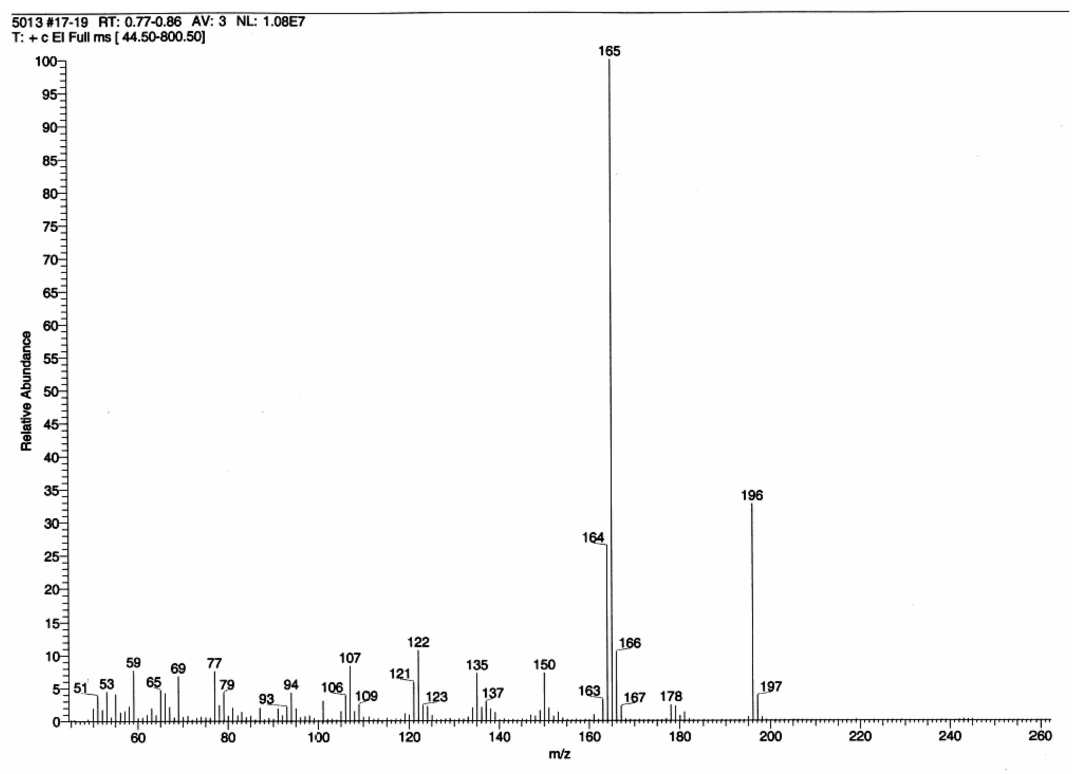

Figure S42. EI-MS spectrum of **6**
